# Vesicle-like nanoparticles extracted from *Pueraria lobata* decoction alleviate colitis by modulating the intestinal microbiota

**DOI:** 10.20517/evcna.2025.134

**Published:** 2026-02-12

**Authors:** Cai-Xiao Liu, Yi-Juan Han, Na Zhao, Qiao-Ning Wang, Run-Run Wan, Ting-Ting Cao, Xi He, Cheng-Hu Hu, Cheng-Biao Hu, Zhang Yuan

**Affiliations:** Xi’an Key Laboratory of Stem Cell and Regenerative Medicine, Institute of Medical Research, Northwestern Polytechnical University, Xi’an 710072, Shaanxi, China.; ^#^These authors contributed equally to this work.

**Keywords:** Plant-derived vesicle-like nanoparticles, *Pueraria lobata Ohwi*, ulcerative colitis, intestinal microorganisms

## Abstract

**Aim:** This study aimed to investigate whether vesicle-like nanoparticles derived from the GeGen decoction (GGD-PDVLNs) represent a key bioactive component responsible for its anti-colitis effects and to elucidate their underlying mechanisms, particularly focusing on gut microbiota modulation.

**Methods:** The GeGen decoction (GGD) was subjected to differential centrifugation following boiling, yielding vesicle-like nanoparticles. Structural analysis confirmed that these nanoparticles have a lipid bilayer and can resist digestion by simulated gastrointestinal fluids. These nanoparticles were administered orally to mice with chronic colitis induced by dextran sulfate sodium to evaluate their therapeutic effects.

**Results:** GGD-PDVLNs effectively mitigated intestinal inflammation by reducing the secretion of pro-inflammatory cytokines [interleukin (IL)-6, IL-1β, tumor necrosis factor-alpha (TNF-α)], elevating levels of the anti-inflammatory cytokine IL-10, alleviating intestinal damage, and enhancing intestinal barrier function, all while exhibiting a favorable biosafety profile. Notably, their therapeutic action depended on gut microbiota modulation. GGD-PDVLNs restored microbial homeostasis, increased microbial diversity, and enriched probiotic populations. In pseudo-germ-free mice, GGD-PDVLNs lost efficacy, confirming microbiota-dependent mechanisms.

**Conclusion:** Vesicle-like nanoparticles are an important active component of GGD. Our findings demonstrate that GGD-PDVLNs significantly ameliorate colonic inflammation through microbiota-dependent mechanisms.

## INTRODUCTION

GeGen is the dried root of the leguminous plant *Pueraria lobata Ohwi*, which is often used in a wide range of applications as a medicine or functional food. It is enriched with diverse bioactive constituents that impart multifaceted pharmacological properties, including hypotensive, hypoglycemic, antitumor, immunomodulatory, anti-inflammatory, and antioxidant activities^[1]^. Owing to its powerful and complex pharmacological effects, GeGen has demonstrated therapeutic potential across multiple diseases, such as cardiovascular disease^[2]^, inflammatory bowel disease (IBD)^[3]^, diabetes^[4]^, alcoholic liver disease^[5]^, and non-alcoholic fatty liver disease^[6]^. Current pharmacological research on GeGen has predominantly focused on small-molecule compounds such as puerarin, which are limited in clinical application due to its poor water solubility, rapid *in vivo* degradation and elimination, and low oral bioavailability^[7,8]^. This imperative drives the need to explore novel bioactive constituents within GeGen that may overcome these pharmacokinetic limitations. A novel active ingredient found in plants, plant-derived vesicle-like nanoparticles (PDVLNs) are bioactive substances with a phospholipid bilayer containing proteins, lipids, DNA, RNA, phytochemicals, and other components that play important roles in intercellular signaling, cargo transport, and disease diagnosis. Their low immunogenicity, easy accessibility, and low cost make them safer and more economical to use as therapeutic agents and drug carriers^[9]^. Moreover, PDVLNs exhibit favorable gastrointestinal (GI) tract stability, enabling oral administration for targeted delivery to colonic inflammatory lesions. The human consumes a variety of PDVLNs in the daily diet, and it has been shown that these nanoparticles are able to enter the circulatory system and deliver effector molecules to cells to modulate cell signaling pathways, which can play an important role in the treatment of disease and the improvement of human health^[10]^. Therefore, we explored the presence of PDVLNs in GeGen and their importance in the pharmacological efficacy of GeGen.

IBD is a chronic inflammatory disorder of the GI tract characterized by continuous mucosal lesions, classified into ulcerative colitis (UC) and Crohn’s disease. Specifically, UC manifests as a continuous inflammatory process that typically originates in the rectum and extends proximally to involve partial or entire colonic segments, with potential peri-appendiceal involvement^[11]^. Current epidemiological data reveal a sustained upward trend in UC incidence worldwide; UC is projected to affect approximately 5 million people worldwide by 2023, with escalating incidence rates placing significant economic pressure on healthcare systems globally^[12]^. GeGen has been widely used in traditional Chinese medicine for UC treatment, and its efficacy has been widely proven. Available evidence suggests that puerarin in GeGen has anti-inflammatory and antioxidant effects, which can alleviate dextran sulfate sodium (DSS)-induced colitis, reduce intestinal damage, inhibit the secretion of inflammatory factors and oxidative stress, and promote the repair of intestinal epithelium^[13,14]^, and also effectively improve the ecological dysregulation of the intestinal flora and alleviate the disorders of amino acid metabolism, and promote the recovery of colitis^[15]^. Additionally, GeGen starch has shown significant regulatory effects on gut microbiota in mice and exerts protective effects against DSS-induced colitis by attenuating colonic inflammation, preserving intestinal barrier integrity, preventing microbial dysbiosis, enhancing short-chain fatty acid (SCFA) production, and inhibiting the nuclear factor kappa B (NF-κB)/interleukin (IL)-1β signaling axis^[16]^. However, the therapeutic potential of GeGen decoction (GGD)-PDVLNs in UC remains unexplored and warrants further investigation.

GeGen-QinLian decoction (GQD), a classical herbal formulation with GeGen as its monarch component, has demonstrated therapeutic efficacy in UC. Our previous study showed that both GQD and its derived PDVLNs (GQD-PDVLNs) significantly ameliorated UC progression. Crucially, further experimental results revealed that the removal of GeGen from the GQD formulation substantially diminished the therapeutic effects of GQD-PDVLNs (detailed mechanistic analysis presented in a companion study currently under peer review). This establishes the indispensable role of GeGen in the fight against colitis.

Herbal ingredients tend to affect the gut microflora and further influence host health by regulating the microecological balance of the gut. GeGen starch can act as a prebiotic to alleviate intestinal inflammation and ischemic stroke by regulating the gut flora^[2]^. Pueraria lobata polysaccharide (PLP1), a novel water-soluble polysaccharide from GeGen, significantly inhibited oxidative stress, regulated lipid metabolism and intestinal flora homeostasis, increased the production of SCFAs, and effectively protected against liver injury in mice with acute alcoholic liver disease^[5]^. Furthermore, a growing body of evidence has demonstrated the role of the microbiome in the pathogenesis of UC^[17]^. Studies of the human gut microbiome have found that the gut microbes of UC patients are significantly different from those of healthy individuals. In most healthy donors, there are four major dominant phyla: Firmicutes, Bacteroidetes, Proteobacteria, and Actinobacteria, with Firmicutes and Bacteroidetes accounting for approximately 90% of the total flora^[18]^. In a metagenomics exploration of the gut microbiota of individuals with UC, there is evidence to support the idea that there is a decrease in both the abundance and diversity of flora, and an increase in the number of harmful flora in the gut of individuals with UC^[19,20]^. Improving UC by modulating microbial composition is a promising therapeutic approach because of the critical role of microorganisms in UC.

In previous studies, PDVLNs have been concentrated in fresh plants^[9,21-24]^, while little research has been done on whether dried Chinese herbs, especially decoctions, contain stable PDVLNs. In this study, structurally stable GGD-PDVLNs were isolated from GGD, proving that GGD-PDVLNs are the main active components of GeGen to exert their medicinal effects. Further studies showed that GGD-PDVLNs could specifically be retained in the inflammatory intestinal site after oral administration and exert a palliative effect on UC through a triple mechanism of action: modulating intestinal inflammation, restoring intestinal barrier function, and maintaining microbiota homeostasis. The present study elucidates that GGD-PDVLNs are the core active ingredients of GGD for the treatment of colitis, and their natural nanocarrier properties achieve synergistic effects and precise delivery of the ingredients, providing a new paradigm for the modernization of this classical compound.

## METHODS

### Preparation of the GGDs

GeGen (ChP 2025, No. 528) was purchased from Huaishuntang Flagship Store in Anhui Province, China. GeGen was crushed and added to double-distilled water (ddH_2_O) according to 1:6 and boiled for 45 min to obtain GGD.

### Isolation of GGD-PDVLNs

GGD was prepared as described above. Decoction was performed by gradient centrifugation (1,000 × *g* for 10 min, 3,000 × *g* for 20 min, 10,000 × *g* for 40 min) at 4 °C to obtain the supernatant, which was then centrifuged at 150,000 × *g* for 70 min at 4 °C. The supernatant obtained after ultra-centrifugation constitutes the vesicle-depleted GeGen decoction (PDVLNs-free GGD), and the GGD-PDVLNs at the bottom of the tube were resuspended in phosphate-buffered saline (PBS) and stored at -80 °C for further use.

### The size distribution and zeta potential analyses

A nanoparticle tracking analysis (NTA) was performed using Zetaview-PMX120-Z (Particle Metrix, Germany) to determine the particle size distribution and particle number of GGD-PDVLNs. Nanoparticle size and zeta potential analyzer (Brookhaven Instruments Corporation, USA) was used to detect the zeta potential of GGD-PDVLNs.

### Transmission electron microscopy

Transmission electron microscopy (TEM) was used to observe the morphological structure of GGD-PDVLNs. The diluted GGD-PDVLNs suspension was deposited onto a carbon-coated copper grid and allowed to stand for 3-5 min to facilitate nanoparticle sedimentation. Excess liquid was blotted with filter paper. The grid was then inverted onto a 2% phosphotungstic acid staining solution for 1-2 min, followed by the removal of residual staining agent. The sample was air-dried at room temperature. Negative-stained specimens were analyzed using a JEM-1400Flash (JEOL, Japan) transmission electron microscope operated at 120 kV. Initial screening and target localization were conducted at low magnification (5,000×), followed by high-resolution image acquisition at 100,000× magnification.

### *In vitro* stability analysis of GGD-PDVLNs

Simulated gastric fluid (SGF) consisted of 80 mM HCl, 35 mM NaCl, and 0.3% pepsin (pH 1.2), and simulated intestinal fluid (SIF) consisted of 15 mM NaOH, 50 mM KH_2_PO_4_, and 1% trypsin (pH 6.8). GGD-PDVLNs (1 mg/mL) were added to PBS, SGF, and SIF, and then incubated at 37 °C for 2 h^[25]^. The stability of GGD-PDVLNs in these solutions was assessed by measuring particle size, particle size distribution, and zeta potential using the methods described previously.

### Determination of Puerarin in GGD-PDVLNs

The GGD-PDVLNs were mixed with 9 times the volume of methanol as described previously^[21,25]^, vortexed and shaken to mix well, centrifuged at 12,000 × *g* for 10 min, and the supernatant was taken and used for high-performance liquid chromatography (HPLC) analysis. An Alliance HPLC e2695 reversed-phase column (Waters, USA) was used for detection. The mobile phase system consisted of acetonitrile and 0.1% aqueous phosphoric acid in the ratio of 12:88, and the chromatographic conditions were set at a constant flow rate of 1.0 mL/min, the ultraviolet (UV) detection wavelength was set at 250 nm, and the temperature of the column was controlled at 30 °C. The sample was injected into the column at 10 μL each time.

### Lipidomic analysis

Lipidomics of GGD-PDVLNs was analyzed using liquid chromatography-tandem mass spectrometry (LC-MS/MS)^[25-27]^. Lipids were extracted from GGD-PDVLNs using organic solvent extraction. Chromatographic separation was performed on an Accucore C30 column (Thermo Scientific, USA) maintained at 40 °C in a column oven. The mobile phase system consisted of: (A) acetonitrile-water (60:40, v/v) with 0.1% formic acid and 10 mM ammonium acetate; and (B) isopropanol-acetonitrile (90:10, v/v) with 0.1% formic acid and 10 mM ammonium acetate. A gradient elution program was applied at a flow rate of 0.35 mL/min, with 5 μL of sample solution injected via an autosampler. Mass spectrometric detection was performed in both positive (ESI+) and negative (ESI-) ion modes, covering a scan range of 114-1,700 m/z.

### Proteomics analysis

Total proteins were extracted from GGD-PDVLNs and quantified using the Bradford method. Following sodium dodecyl sulfate-polyacrylamide gel electrophoresis (SDS-PAGE) separation and excision of target protein bands, in-gel enzymatic digestion was performed. The resulting peptides were lyophilized and stored at -80 °C until LC-MS/MS analysis. The mobile phases of the LC-MS/MS system consisted of liquid A (0.1% formic acid aqueous solution) and liquid B (0.1% formic acid/80% acetonitrile). The lyophilized samples were dissolved in 10 µL of solution A and centrifuged at 14,000 × *g* for 20 min at 4 °C, and the supernatant was injected with a sample volume of 200 ng. A Vanquish Neo Ultra High Performance Liquid Chromatography (UHPLC) system (Thermo Scientific, USA) equipped with a C18 pre-column (5 mm × 300 μm, 5 μm) and an analytical column (150 µm × 15 cm, 2 μm), the column temperature was set at 50 °C. The mass spectrometry analysis was performed on an Orbitrap Astral system (Thermo Scientific, USA) equipped with an ESI ion source and a spray voltage of 2.0 kV. The primary mass spectrometry scanning range was set at 380-980 m/z, and the secondary mass spectrometry acquisition range was set at 150-2,000 m/z. The data acquisition was performed in data-independent acquisition (DIA) mode.

### Animals and cell culture

Wild-type C57BL/6J male mice aged 6 to 8 weeks (22 to 24 g) were purchased from Beijing Spaf Biotechnology Co., Ltd. (Beijing, China). Mice were housed in individually ventilated cages with free access to food and water at 23 ± 2 °C, 55% ± 5% humidity, and a light/dark cycle of 12 h. The animal experimental protocols were approved by the Medical and Animal Ethics Committee of Northwestern Polytechnical University, Xi’an, Shaanxi, China (Approval No. 202501087).

Human epithelial colorectal adenocarcinoma Caco-2 cell lines were cultured in Minimal Essential Medium (MEM, Thermo Scientific, USA) enriched with 20% fetal bovine serum (FBS) and 100 U/mL penicillin-streptomycin. Raw 264.7 murine macrophages were cultured in Dulbecco’s Modified Eagle Medium (DMEM, Thermo Scientific, USA) containing with 10% FBS and 100 U/mL penicillin-streptomycin. The above cells were all cultured in a humidified 5% CO_2_ at 37 °C.

### Distribution of GGD-PDVLNs *in vivo*

GGD-PDVLNs were diluted to 1 mg/mL and incubated with 1,1’-dioctadecyl-3,3,3’,3-tetramethylindotricarbocyaine iodide (DiR) dye at a final concentration of 10 μM, followed by protected incubation at 37 °C for 30 min. The mixture was then subjected to ultracentrifugation at 170,000 × *g* for 2 h at 37 °C. After discarding the supernatant, the pellet was resuspended in an appropriate volume of PBS to obtain DiR-labeled GGD-PDVLNs^[28]^.

Healthy and colitis mice were administered orally with DiR-labeled GGD-PDVLNs. Mice were anaesthetized at predetermined times (0.5, 1, 2, 3, 6, 12, 24 h), and fluorescence signals were acquired using the IVIS Lumina LT Series III imaging system (Perkin Elmer Instruments Co., Ltd, USA) to determine the distribution of GGD-PDVLNs in mice. Then, at designated time points (3, 6, 12, 24 h), mice were euthanized, followed by the collection of the stomach, entire GI tract, and major organs (heart, liver, spleen, lungs, kidneys). The distribution of fluorescent signals was then systematically examined across all harvested tissues.

### Therapeutic effects of GGD-PDVLNs in DSS-induced colitis

After 7 days of acclimatization, the mice were randomly divided into three groups: the healthy control group, DSS group, and DSS + GGD-PDVLNs group. Colitis was induced by adding 2% DSS (MW 36,000-50,000 kDa, MP Biochemicals, USA) to the drinking water of the mice for 7 consecutive days, with fresh DSS solution replaced every other day. Meanwhile, the colitis mice were treated with PBS or GGD-PDVLNs (15 mg/kg) daily for seven days. The mice were monitored, scored and recorded daily for weight changes, fecal consistency, and fecal bleeding^[29,30]^. On day 5 of treatment, mouse feces were collected in a sterile environment and used for gut flora analysis. After 7 days, the mice were sacrificed, and the colon was collected for pathological and inflammatory analysis. Major organs (heart, liver, spleen, lungs, and kidneys) were harvested from euthanized mice and processed for hematoxylin and eosin (H&E) staining to assess the potential toxicity and biocompatibility of GGD-PDVLNs.

### Therapeutic effects of GGD and PDVLNs-free GGD in DSS-induced colitis

As described above, the colitis mouse model was established and subsequently treated for seven consecutive days with PBS, GGD (37 mg/kg), and PDVLNs-free GGD (37 mg/kg). All treatments were administered daily via gavage. Following the treatment period, mice were euthanized and colon tissues were collected for subsequent analysis.

### H&E staining analyses

The mouse colon and different organs were fixed in 4% paraformaldehyde for 24 h and then paraffin-embedded at room temperature. Tissue sections, with a thickness of 6 μm, were subsequently stained with H&E, and images were acquired using a BX53 (Olympus Corporation, Japan) microscope.

### Enzyme-linked immunosorbent assay

Colon tissue samples were homogenized in ice-cold PBS at a 1:9 (w/v) ratio. The homogenate was subsequently centrifuged at 12,000 *g* for 5 min at 4 °C to pellet cellular debris. The contents of IL-1β, tumor necrosis factor-alpha (TNF-α), IL-6, and IL-10 in colon tissue were determined using commercial enzyme-linked immunosorbent assay (ELISA) kits (NeoBioscience Technology Co., Ltd., China) according to the manufacturer’s instructions.

### RNA extraction and real-time reverse transcription-polymerase chain reaction assay

Total RNA was extracted from colon tissues using FreeZol Reagent (Nanjing Vazyme Biotech Co., Ltd, China) according to the manufacturer’s instructions. First-strand cDNA was synthesized using the HiScript IV All-in-One Ultra RT SuperMix for qPCR kit (Nanjing Vazyme Biotech Co., Ltd, China). Real-time reverse transcription-polymerase chain reaction (RT-qPCR) was performed to obtain the relative messenger RNA (mRNA) expression of inflammatory factors using the Taq Pro Universal SYBR qPCR (quantitative polymerase chain reaction) Master Mix kit (Nanjing Vazyme Biotech Co., Ltd, China). The primer sequences can be found in Supplementary Table 1.

### Immunofluorescence staining

Optimal Cutting Temperature (OCT) compound-embedded tissues were sectioned at a thickness of 20 μm, washed with PBS to remove the embedding agent, and blocked by incubating with goat serum for 1 h at 4 °C. Subsequently, the samples were incubated with rabbit anti-mouse occludin or zonula occludens (ZO)-1 antibody (Abcam plc, 1:100 dilution) overnight at 4 °C. Then, they were incubated with Alexa Fluor 488-conjugated goat anti-rabbit secondary antibody [Yeasen Biotechnology (Shanghai) Co., Ltd., 1:400 dilution] for 1 h at room temperature, and the coverslips were mounted with an anti-fluorescence quenching mounting medium containing DAPI^[31]^. Image acquisition was performed using an FV3000 laser confocal microscope (Olympus Corporation, Japan).

### Hemolysis rate analysis

Fresh blood was collected from mice using anticoagulation tubes, diluted with nine volumes of PBS, and centrifuged at 300 × *g* for 10 min to collect erythrocytes. This process was repeated three times. Subsequently, a 10% erythrocyte suspension was prepared by adding nine volumes of PBS, and gradient dilutions of GGD-PDVLNs (1.6, 0.8, 0.4, 0.2, 0.1, 0.05, 0.025 mg/mL), ddH_2_O, and PBS were added, respectively. The mixture was incubated for 1 h at 37 °C and then centrifuged at 300 × *g* for 10 min to collect the supernatant, and the absorbance was measured at 540 nm using a microplate reader.

### Cytotoxicity assay

RAW 264.7 cells and Caco-2 cells were seeded into 96-well plates at a density of 2 × 10^4^ cells/well. After adhesion, the medium was replaced with fresh medium containing a gradient dilution of GGD-PDVLNs. After 24 h, the medium was removed, cells were washed once with PBS, and fresh medium containing 10% CCK-8 solution was added. Following 1 h of incubation, the absorbance was measured at 450 nm using a microplate reader.

### Microbiome analysis

Fecal samples for microbiome analysis were collected using a standardized non-invasive protocol. Specifically, on day 5 of treatment, individual mice were placed in sterile, separate cages. Fresh fecal pellets were collected immediately after defecation, rapidly frozen in liquid nitrogen, and stored at -80 °C until DNA extraction. Mouse fecal samples were transported on dry ice to Novogene Co. for analysis. Briefly, DNA was extracted from the samples, then separated on an agarose gel. An Agilent 5400 Automated Capillary Electrophoresis System (Agilent Technologies Inc, USA) was used for DNA quality control and polymerase chain reaction (PCR) targeting the V4 region of the bacteria (515F, 5’-GTGCCAGCMGCCGCGGGTAA-3’, 806R, 5’- GGACTACHVGGGTWTCTAAT-3’)^[32]^. The PCR products were purified using magnetic beads and then mixed in equal amounts according to their concentrations. After thorough mixing, the pooled products were analyzed by electrophoresis, and the target DNA bands were excised for recovery. Following quality control verification of the DNA samples, the PCR amplicons were pooled, purified, and processed for library construction. The constructed library was quantified using Qubit and qPCR, then sequenced on the Illumina platform.

### Gut microbes uptake GGD-PDVLNs assay

*In vitro* uptake assay, fresh fecal samples were collected from mice and homogenized in PBS. The homogenate was subjected to gradient centrifugation to isolate the intestinal microbiota. The collected bacterial fraction was then co-incubated with PKH-26-labeled GGD-PDVLNs at 37 °C for 3 h. After incubation, the bacterial suspensions were carefully mounted on glass slides and imaged using a laser scanning confocal microscope (OLYMPUS FV3000, Japan).

### Pseudo germ-free mouse model establishment

A pseudo-germ-free model was constructed based on our previous studies. In brief, mice were given an antibiotic cocktail including ampicillin sodium salt (1 g/kg), neomycin sulfate (1 g/kg), vancomycin hydrochloride (0.5 g/kg), and metronidazole (0.125 g/kg) for five consecutive days to deplete the gut microbiota before inducing colitis^[33]^.

### Statistical analysis

The Graphic Abstract was created in BioRender. Statistical analysis was performed by using the GraphPad Prism 9.0 software. The Shapiro-Wilk test was used to determine whether the data followed a normal distribution. Data were presented as mean ± Standard Error of the Mean (SEM). Statistical significance was assessed using one-way and two-way analyses of variance (ANOVAs). All results were considered statistically significant at *P* < 0.05 (^*^P < 0.05; ^**^P < 0.01; ^***^P < 0.001), and ns means no significance.

## RESULTS

### Oral administration of GGD relieved DSS-induced UC

**Figure 1:**
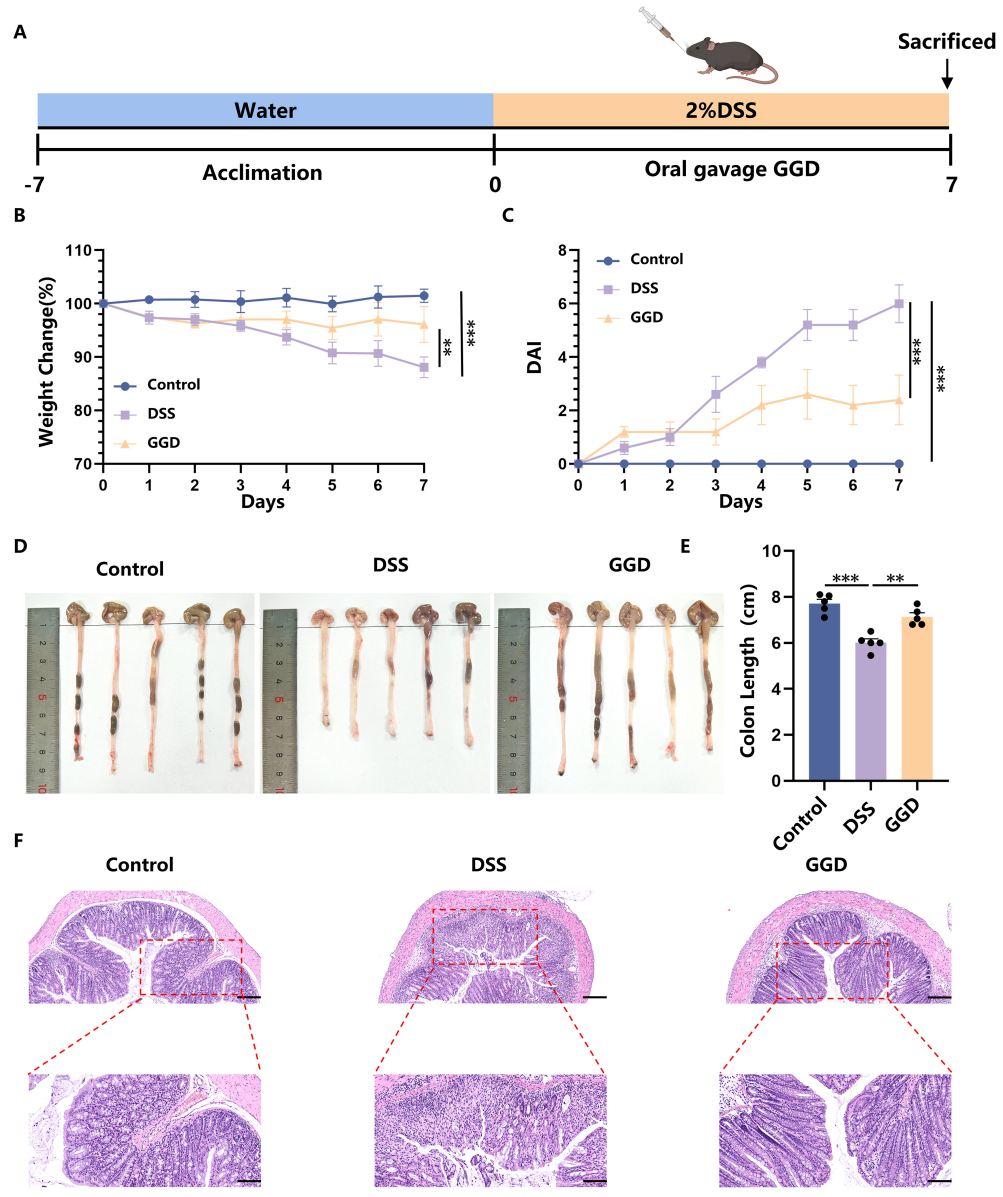
Oral administration of GGD alleviates DSS-induced UC. (A) Protocol for DSS-induced colitis and GGD administration, created in BioRender. Yijuan, H. (2026) https://BioRender.com/68i1gyz; (B) Change in body weight, as a percentage of the weight on day zero; (C) DAI score, DAI score = weight loss score (< 1%-0, 1%-5%-1, 6%-10%-2, 11%-20%-3, > 20%-4) + fecal status score (normal-0, soft stools-1, loose stools-2, watery stools-3) + bleeding condition score (normal-0, slight bleeding-1, significant bleeding-2, heavy bleeding-3); (D and E) Representative images of colons and average colon length from different groups, *n* = 5; (F) H&E-stained colon sections (100 and 50 μm, *n* = 3). Data are shown as means ± SEM. Bar graphs were analyzed using one-way ANOVA, while line graphs were analyzed using two-way ANOVA. ^**^P < 0.01; ^***^P < 0.001. GGD: GeGen decoction; DSS: dextran sulfate sodium; UC: ulcerative colitis; DAI: disease activity index; H&E: hematoxylin and eosin; SEM: standard error of the mean; ANOVA: analysis of variance; P: probability value.

In this study, we first investigated the therapeutic potential of GGD in alleviating UC symptoms in a mouse model [Figure 1A]. Administration of DSS induced severe colitis in mice, as evidenced by significant weight loss [Figure 1B], diarrhea, and bloody stools, which collectively resulted in markedly increased disease activity index (DAI) scores [Figure 1C]. Furthermore, oral administration of GGD effectively ameliorated colitis symptoms. GGD-treated mice exhibited better weight maintenance, retaining approximately 88% of their initial body weight, along with significantly reduced DAI scores [Figure 1B and C]. Colon length is also one of the key parameters to assess the severity of inflammation, which was significantly shorter in DSS-treated mice (6.18 ± 0.49 cm) compared to controls (7.67 ± 0.35 cm), reflecting the characteristic intestinal inflammation. Notably, GGD treatment preserved colon length (7.26 ± 0.44 cm), further supporting its protective effect against DSS-induced colitis [Figure 1D and E]. In addition to the above phenotypes, the alleviation of pathological damage to intestinal tissues is also an important indicator of UC treatment, including the restoration of normal crypt structure and goblet cells, intact epithelial state and reduced inflammatory infiltration. While cupular cells secrete mucin, and mucus forms a barrier to limit the direct interaction between microorganisms and the epithelial cells^[34]^, protecting the intestinal epithelial cells. The intestinal morphology and structure of the mice in the control group, with intact epithelial cells and crypt structures, indicated that the degree of intestinal tissue damage in the DSS group of mice was significantly increased, and the crypt structure appeared to be severely damaged, with a significant reduction in the number of its characteristic cup-shaped cells. Pathological thickening of the submucosa was also observed, accompanied by a large number of aggregated and infiltrated inflammatory cells. After oral administration of GGD, the intestinal damage in mice was significantly alleviated [Figure 1F]. These results suggest that GGD can effectively alleviate the intestinal damage caused by DSS.

### Isolation and characterization of GGD-PDVLNs

**Figure 2:**
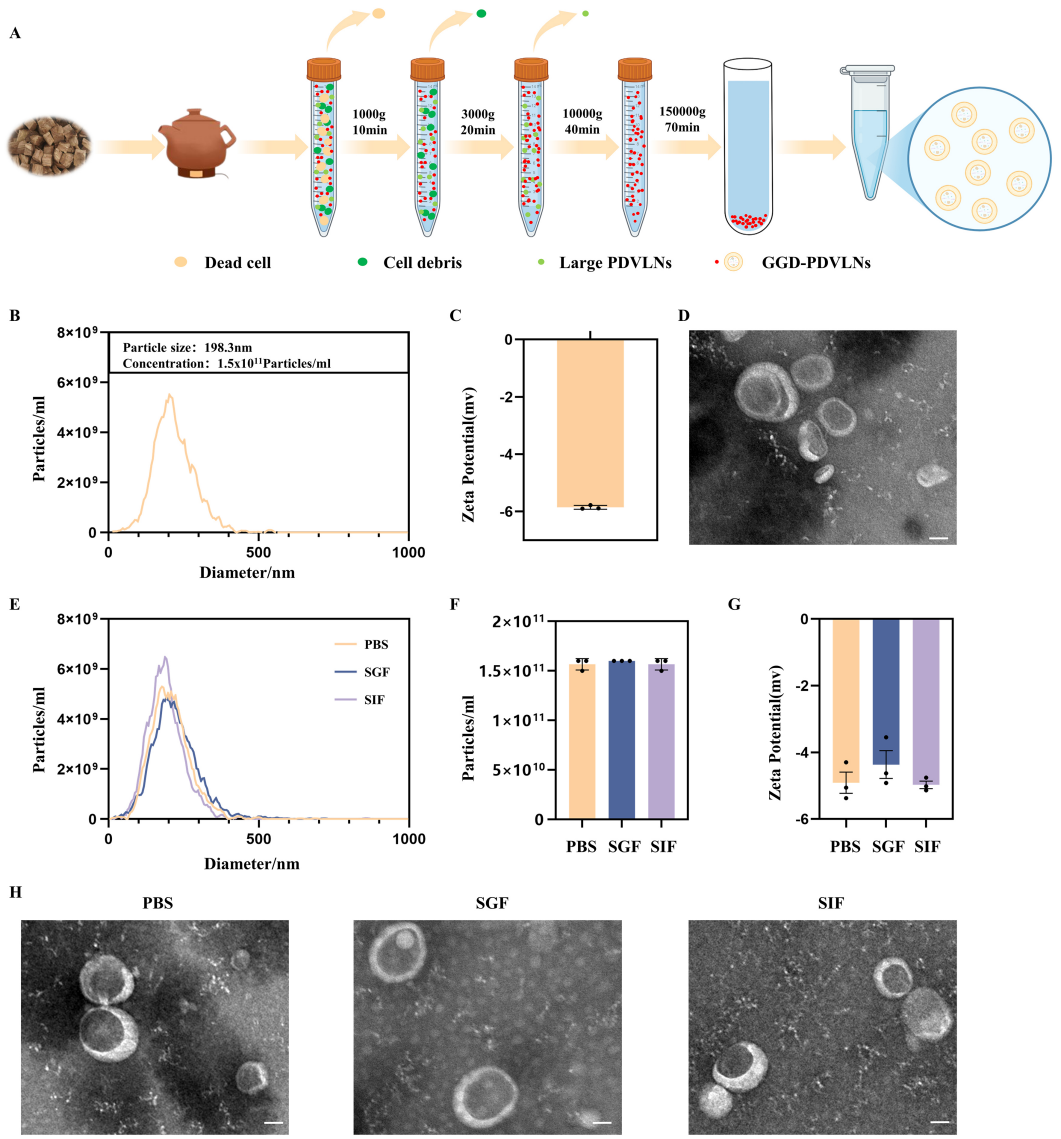
Isolation and characterization of GGD-PDVLNs. (A) The isolation and purification scheme of GGD-PDVLNs, created in BioRender. Yijuan, H. (2026) https://BioRender.com/01oosim; (B) NTA analysis; (C) Zeta potential; (D) TEM images (50 nm); (E-H) Particle size distribution, particle number change, zeta potential change, TEM images of GGD-PDVLNs after 2 h incubation in PBS, SGF, SIF. Data are shown as means ± SEM, *n* = 3. GGD-PDVLNs: GeGen decoction-derived vesicle-like nanoparticles; NTA: nanoparticle tracking analysis; TEM: transmission electron microscopy; PBS: phosphate-buffered saline; SGF: simulated gastric fluid; SIF: simulated intestinal fluid; SEM: standard error of the mean.

To identify the bioactive components underlying the therapeutic efficacy of GGD, we isolated GGD-PDVLNs through a sequential purification protocol involving differential centrifugation followed by ultracentrifugation, as schematized in Figure 2A. NTA demonstrated the mono-dispersed particles with a mean hydrodynamic diameter of 198.3 nm at a concentration of 1.5 × 10^11^ particles/mL per mg of GGD-PDVLNs [Figure 2B]. Zeta potential measurements confirmed a negative surface charge (-5.89 ± 0.01 mV), consistent with the characteristic electronegativity of phospholipid bilayers [Figure 2C]. TEM results showed that the GGD-PDVLNs were cup-shaped structures under the microscope with an obvious lipid bilayer, similar to the structure of animal exosomes, and their sizes were consistent with those of the NTA test [Figure 2D]. The complex GI environment poses a significant challenge to the oral delivery of many therapeutic agents. To evaluate the suitability of GGD-PDVLNs as an oral formulation, we first assessed their resistance to GI digestion. GGD-PDVLNs were incubated in PBS, SGF, and SIF at 37 °C for 2 h. NTA revealed that both the particle size and concentration of GGD-PDVLNs remained stable after exposure to SGF and SIF [Figure 2E and F]. The zeta potential of GGD-PDVLNs exhibited a slight decrease in absolute value in SGF, likely due to pH effects, but overall maintained stability. In contrast, the zeta potential in SIF showed negligible variation [Figure 2G]. Further characterization by TEM confirmed that GGD-PDVLNs did not change before and after digestion, and the structure was intact [Figure 2H]. These results indicate that GGD-PDVLNs exhibit robust resistance to GI digestion, supporting their potential to remain intact during transit through the GI tract and effectively reach the site of intestinal inflammation following oral administration.

### The important active components of GGD-PDVLNs

GeGen is a rich source of puerarin, a bioactive isoflavone with significant therapeutic potential in both *in vitro* and *in vivo* models of cardiovascular diseases and colitis, and an important role in modulating inflammation^[35,36]^. The qualitative and quantitative analysis of puerarin in GGD-PDVLNs was carried out using HPLC. Our HPLC analysis revealed that GGD-PDVLNs contain multiple bioactive compounds, with puerarin being the most abundant constituent (35.4 ± 1.2 μg/mg of GGD-PDVLNs; [Supplementary Figure 1A and B].

PDVLNs are rich in lipids, which have important physiological functions. In this study, the lipids in GGD-PDVLNs were identified by LC-MS/MS based on a non-targeted approach. Under positive ion mode, a total of 1,455 lipid compounds were identified, mainly glycerol lipids, glycerophospholipids and sphingolipids, and the lipid subclasses mainly included triglycerides (TG, 33.33%), diglycerides (DG, 14.78%), ceramides (Cer, 9.35%), phosphatidylcholines (PC, 7.9%), hexosylceramides (hex1Cer, 5.36%), and phosphatidylethanolamines (PE, 3.78%) [Supplementary Figure 1C and D]. Lipids in the samples were annotated using the Kyoto Encyclopedia of Genes and Genomes (KEGG) pathway database^[37]^. KEGG analysis showed that lipids in GGD-PDVLNs were mainly associated with a variety of cellular processes (transport and catabolism, cell growth and death), environmental information processing (signaling molecules and interactions, signal transduction), metabolism (lipid metabolism, glycan biosynthesis and metabolism, carbohydrate metabolism, amino acid generation, *etc*.), and multiple organic systems (sensory system, nervous system, immune system, endocrine system, *etc*.) [Supplementary Figure 1E].

We next characterized the protein composition of GGD-PDVLNs through comprehensive proteomic analysis. Mass spectrometry identified 3,192 distinct proteins, with subcellular localization analysis revealing a diverse distribution: cytoplasmic proteins constituted the largest proportion (22.09%), followed by nuclear proteins (15.94%), cell membrane-associated proteins (13.48%), mitochondrial proteins (8.22%), and extracellular proteins (7.8%) [Supplementary Figure 1F]. In order to understand the biological pathways in which the proteins are mainly involved, we performed KEGG pathway enrichment analysis. The KEGG results indicated that the proteins in GGD-PDVLNs are essential for carbohydrate metabolism pathways, amino acid metabolism pathways, translation, folding, and degradation [Supplementary Figure 1G].

### *In vivo* distribution of GGD-PDVLNs

To investigate the biodistribution of GGD-PDVLNs in mice, fluorescence imaging was performed using the IVIS imaging system after oral administration of DiR-labeled GGD-PDVLNs to healthy mice and UC model mice. As shown in [Supplementary Figure 2A], the fluorescence signals in both groups of mice exhibited a trend of increasing and then gradually decreasing. In control mice, peak fluorescence intensity was observed within 2 h post-administration, predominantly localized in the gastric and intestinal regions, and signal intensity gradually decreased between 3 and 24 h, with complete clearance by 24 h. In UC model mice, the fluorescence signal was significantly stronger than in control mice, indicating a greater propensity of GGD-PDVLNs to accumulate in inflamed intestinal tissue. At 12 h, the fluorescent signals were very weak in control mice. In contrast, strong fluorescent signals were still retained in colitis model mice, and the signals of GGD-PDVLNs also almost disappeared in UC model mice at 24 h. To further examine the distribution of DiR-labeled GGD-PDVLNs in tissues and organs of mice, the mice were sacrificed at a specific time point after oral administration. The main tissues, organs, and GI tract of the mice were collected, and fluorescence imaging was performed using the IVIS imaging system. As shown in [Supplementary Figure 2B], there was almost no fluorescence signal in the heart, liver, spleen, lungs and kidneys in either control mice or UC mice. In contrast, strong fluorescence signals were retained in the stomach and intestines. Similar to the results of [Supplementary Figure 2A], the intensity of fluorescent signals in the intestines of UC mice was also higher than that of control mice. These results suggest that GGD-PDVLNs can preferentially accumulate in colitis tissues.

### GGD-PDVLNs serve as the primary active components of GGD in alleviating colitis

**Figure 3:**
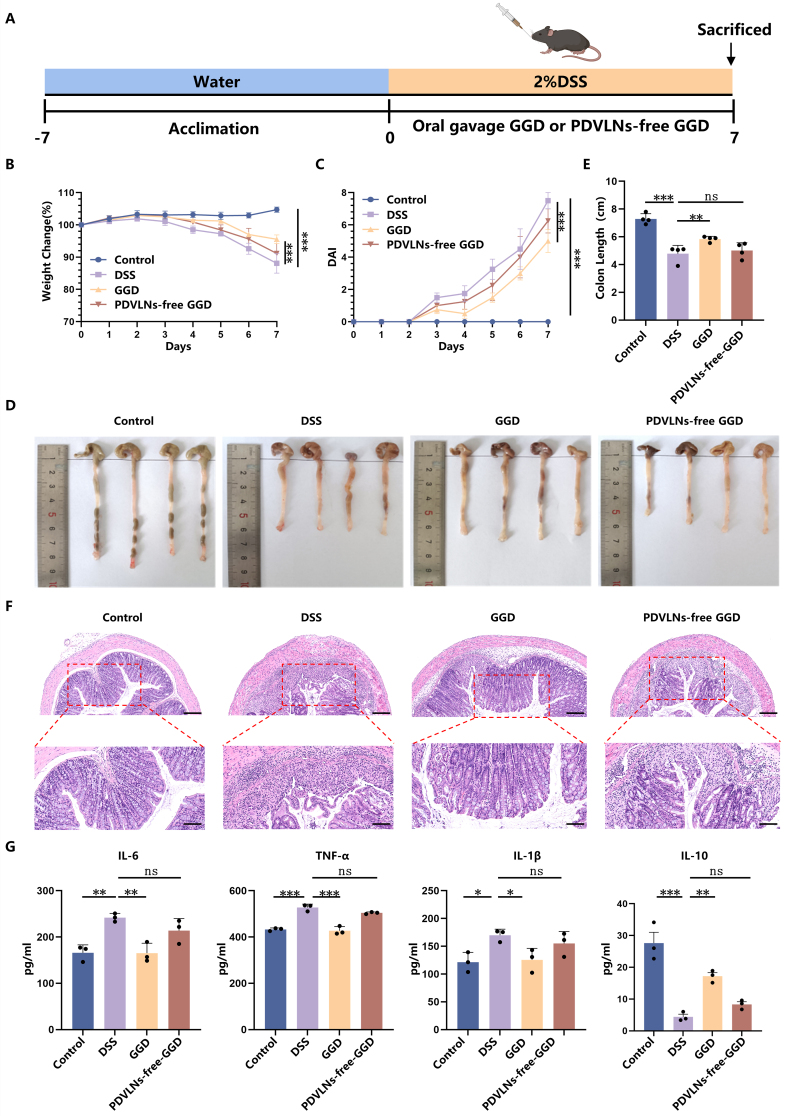
GGD-PDVLNs serve as the primary active components of GGD in alleviating colitis. (A) Protocol for DSS-induced colitis and GGD or PDVLNs-free GGD administration, created in BioRender. Yijuan, H. (2026) https://BioRender.com/zb6hfk8; (B) Change in body weight, as a percentage of the weight on day zero; (C) DAI score; (D and E) Representative images of colons and average colon length from different groups, *n* = 4; (F) H&E-stained colon sections (100 and 50 μm, *n* = 3); (G) ELISA testing the expression profiles of IL-6, IL-1β, TNF-α and IL-10 in colon tissue, *n* = 3. Data are shown as means ± SEM. Bar graphs were analyzed using one-way ANOVA, while line graphs were analyzed using two-way ANOVA. ^*^P < 0.05; ^**^P < 0.01; ^***^P < 0.001. GGD: GeGen decoction; GGD-PDVLNs: GeGen decoction-derived vesicle-like nanoparticles; PDVLNs: plant-derived vesicle-like nanoparticles; DSS: dextran sulfate sodium; DAI: disease activity index; H&E: hematoxylin and eosin; ELISA: enzyme-linked immunosorbent assay; IL: interleukin; TNF-α: tumor necrosis factor alpha; SEM: standard error of the mean; ANOVA: analysis of variance; ns: no significance.

Our previous study demonstrated that GGD exhibited significant therapeutic efficacy against UC. To confirm that PDVLNs are the primary bioactive constituents responsible for GGD’s pharmacological activity, we conducted depletion experiments by removing PDVLNs from GGD using ultracentrifugation. Subsequent therapeutic evaluation compared the effects of intact GGD versus PDVLN-free GGD through oral administration in UC model mice [Figure 3A]. Our results showed that, consistent with [Figure 1B and C], administration of GGD significantly alleviated weight loss and elevated DAI scores in mice [Figure 3B and C], demonstrating effective mitigation of colitis symptoms. Notably, PDVLN-free GGD largely lost its therapeutic capacity, with treated mice exhibiting comparable weight loss patterns [Figure 3B] and DAI progression [Figure 3C] to the DSS group. The colon was significantly shorter in the DSS group (4.78 ± 0.53 cm) compared with the control group (7.29 ± 0.32 cm), and the colon length was significantly increased after the administration of GGD (5.83 ± 0.17 cm). However, the administration of PDVLN-free GGD showed limited restorative capacity, achieving only partial recovery (5.01 ± 0.48 cm; Figure 3D and E). H&E staining similarly showed that GGD treatment substantially mitigated colonic damage. However, PDVLN-free GGD exhibited incomplete therapeutic efficacy, with residual pathological features including persistent inflammatory cell infiltration, submucosal thickening, and disrupted crypt regeneration [Figure 3F]. This differential therapeutic performance between intact and PDVLN-free GGD collectively demonstrates that PDVLNs serve as the primary bioactive constituents mediating the anti-colitis effects of GGD. The pathogenesis of UC involves massive infiltration of inflammatory cells into intestinal tissues, leading to elevated production of nonspecific inflammatory mediators, particularly pro-inflammatory cytokines (IL-6, IL-1β, and TNF-α)^[38]^. In contrast, IL-10, a pleiotropic cytokine with potent anti-inflammatory properties, plays a crucial regulatory role by suppressing the expression of these pro-inflammatory mediators^[39]^. To elucidate the anti-inflammatory effects of GGD, we systematically analyzed both protein and mRNA expression levels of key inflammatory factors (IL-6, IL-1β, TNF-α, and IL-10) in intestinal tissues. DSS treatment resulted in a significant increase in the levels of pro-inflammatory cytokines (IL-6, TNF-α, IL-1β), while anti-inflammatory cytokine IL-10 was significantly decreased, indicating a more severe inflammatory infiltration. The protein levels of IL-6, TNF-α, and IL-1β were significantly decreased, and IL-10 expression was significantly increased after treatment with GGD. However, the modulatory effect of PDVLN-free GGD on inflammatory factors was markedly diminished [Figure 3G]. Supplementary Figure 3 presents the mRNA levels of inflammatory factors in the mouse intestines as determined by RT-qPCR, revealing a consistent trend between mRNA and protein expression profiles. These findings collectively indicate that GGD exerts a significant inhibitory effect on intestinal inflammation in mice.

### Oral administration of GGD-PDVLNs alleviates DSS-induced UC

**Figure 4:**
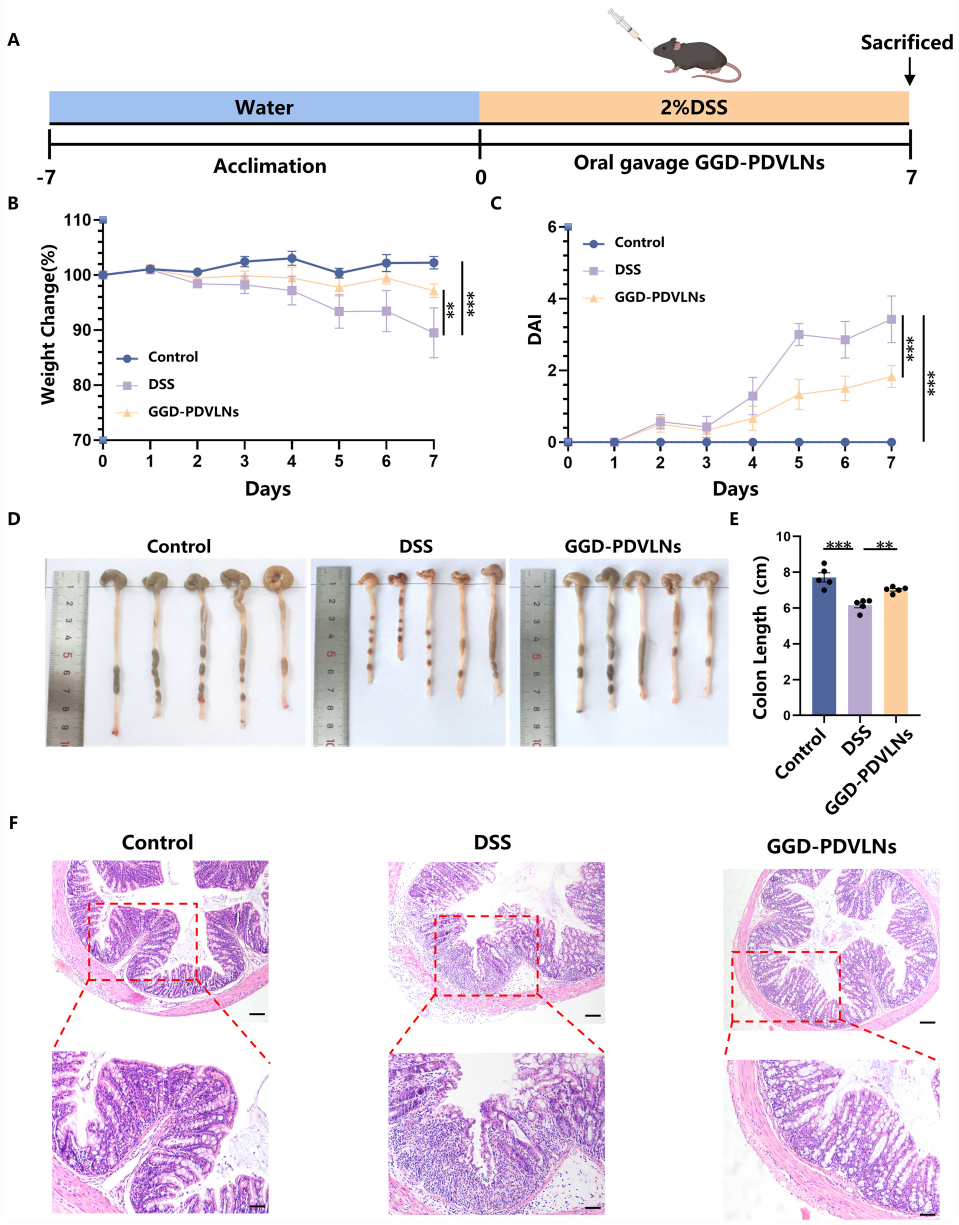
Oral administration of GGD-PDVLNs alleviates DSS-induced UC. (A) Protocol for DSS-induced colitis and GGD-PDVLNs administration, created in BioRender. Yijuan, H. (2026) https://BioRender.com/vhtvulv; (B) Change in body weight, as a percentage of the weight on day zero; (C) DAI score; (D and E) Representative images of colons and average colon length from different groups, *n* = 5; (F) H&E-stained colon sections (100 and 50 μm, *n* = 3). Data are shown as means ± SEM. Bar graphs were analyzed using one-way ANOVA, while line graphs were analyzed using two-way ANOVA. ^**^P < 0.01; ^***^P < 0.001. GGD-PDVLNs: GeGen decoction-derived vesicle-like nanoparticles; DSS: dextran sulfate sodium; UC: ulcerative colitis; DAI: disease activity index; H&E: hematoxylin and eosin; SEM: standard error of the mean; ANOVA: analysis of variance.

To evaluate the therapeutic potential of GGD-PDVLNs in UC, we established a DSS-induced UC model and administered either PBS or GGD-PDVLNs [Figure 4A]. Oral administration of GGD-PDVLNs significantly alleviated DSS-induced weight loss and the increase in DAI score in mice [Figure 4B and C]. PBS-treated mice showed progressive weight loss, declining to 90% of initial body weight by day 7. In contrast, GGD-PDVLN treatment maintained body weight at 97% [Figure 4B], which exhibited similar therapeutic effects to GGD. This result further suggests that GGD-PDVLNs are the effective components in GGD. DSS induced severe colon shortening (6.16 ± 0.30 cm *vs*. Control: 7.71 ± 0.52 cm). Strikingly, GGD-PDVLN treatment significantly restored colon length to 7.02 ± 0.15 cm [Figure 4D and E], further confirming its potent anti-colitis activity. H&E staining showed significant inflammatory cell infiltration in the intestines of mice in the DSS group, with disruption of the intestinal epithelial barrier, loss of crypt structure, and decreased goblet cell numbers. In contrast, the intestines of mice orally administered GGD-PDVLNs were similar to those of the control group, with intact crypt structures, a significant increase in goblet cells, and no obvious inflammatory cell infiltration, indicating that GGD-PDVLNs effectively alleviated colitis in mice [Figure 4F].

**Figure 5:**
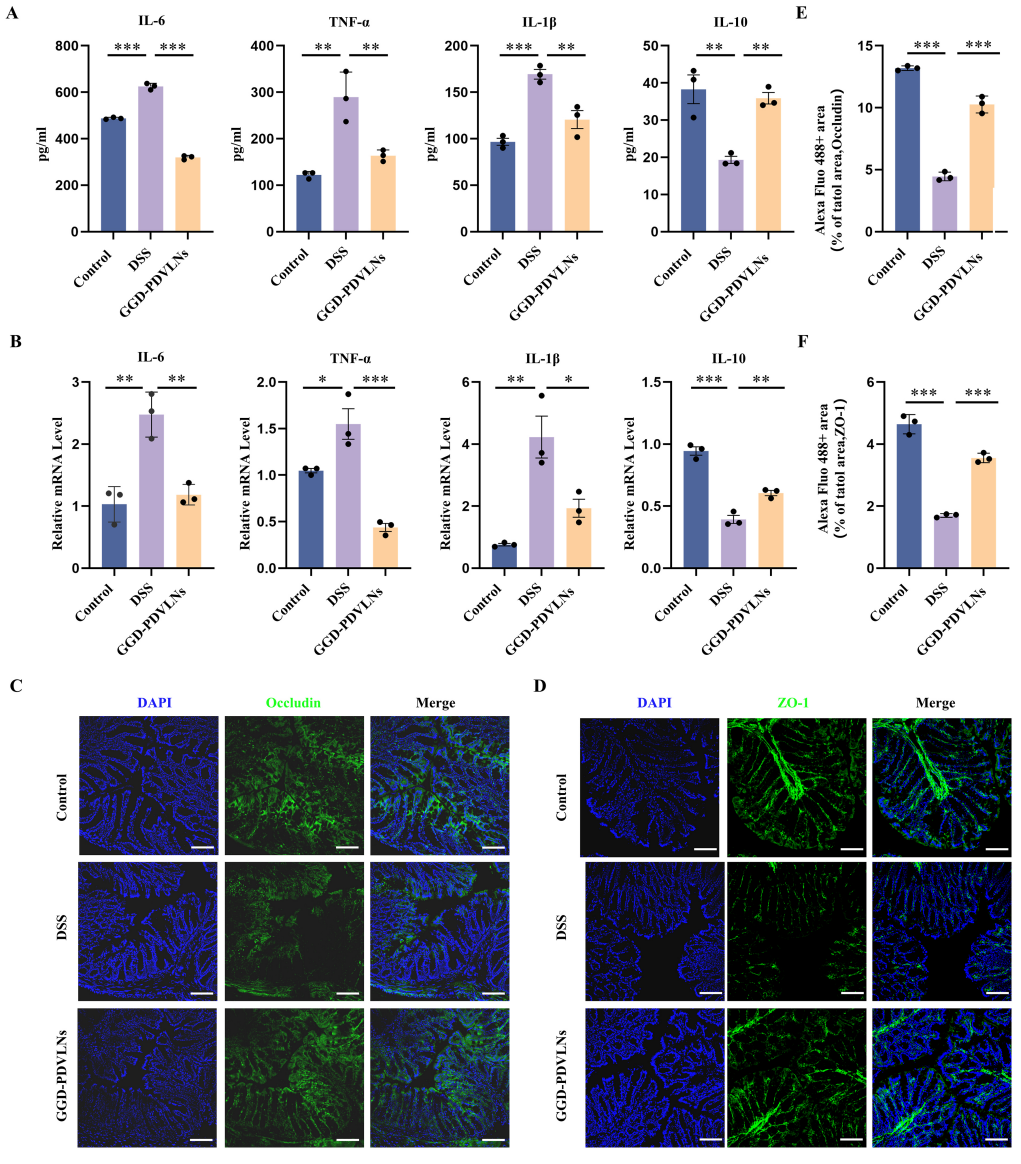
GGD-PDVLNs reduce intestinal inflammation and restore the intestinal epithelial barrier. (A) ELISA testing the expression profiles of IL-6, IL-1β, TNF-α, and IL-10 in colon tissue; (B) RT-qPCR detecting the levels of IL-6, IL-1β, TNF-α and IL-10 in colon tissue; (C and E) Fluorescence images of occludin in colon samples and quantitative statistics of relative fluorescence area; (D and F) Fluorescence images of ZO-1 in colon samples and quantitative statistics of relative fluorescence area; scale bar = 100 μm. *n* = 3. Data are shown as means ± SEM. Bar graphs were analyzed using one-way ANOVA. ^*^P < 0.05; ^**^P < 0.01; ^***^P < 0.001. GGD-PDVLNs: GeGen decoction-derived vesicle-like nanoparticles; ELISA: enzyme-linked immunosorbent assay; RT-qPCR: real-time quantitative polymerase chain reaction; IL: interleukin; TNF-α: tumor necrosis factor alpha; ZO-1: zonula occludens-1; SEM: standard error of the mean; ANOVA: analysis of variance.

GGD-PDVLNs exhibited comparable anti-inflammatory efficacy to GGD, demonstrating dual regulatory effects through significant suppression of pro-inflammatory IL-6, TNF-α, and IL-1β secretion and restoration of anti-inflammatory IL-10 production in colon tissues [Figure 5A and B]. In addition to reducing inflammation, repair of the intestinal barrier is one of the most important indicators of UC relief. In UC, intestinal barrier function is reduced due to damage to epithelial cell tight junctions and increased intestinal permeability. The first tight junction protein identified was ZO-1, followed by two related proteins, ZO-2 and ZO-3, which are peripheral membrane proteins^[40]^. These findings were followed by the discovery of occludin and claudin, four transmembrane tight junction proteins encoded by 27 genes in mammals. To investigate the ability of GGD-PDVLNs to repair the intestinal barrier, ZO-1 and occludin were measured using immunofluorescence (IF). ZO-1 [Figure 5C] and occludin [Figure 5D] proteins were significantly reduced in the intestines of the DSS group compared to the control group, consistent with the evident pathological damage observed in the intestinal tissues of DSS-treated mice shown in Figure 4F. In contrast, treatment with GGD-PDVLNs markedly attenuated this reduction, restored the integrity of the intestinal epithelial barrier, and reduced antigen-induced epithelial erosion. Quantitative analyses are presented in [Figure 5E and F].

### GGD-PDVLNs modulate gut microbiota diversity and increase the abundance of probiotics

**Figure 6:**
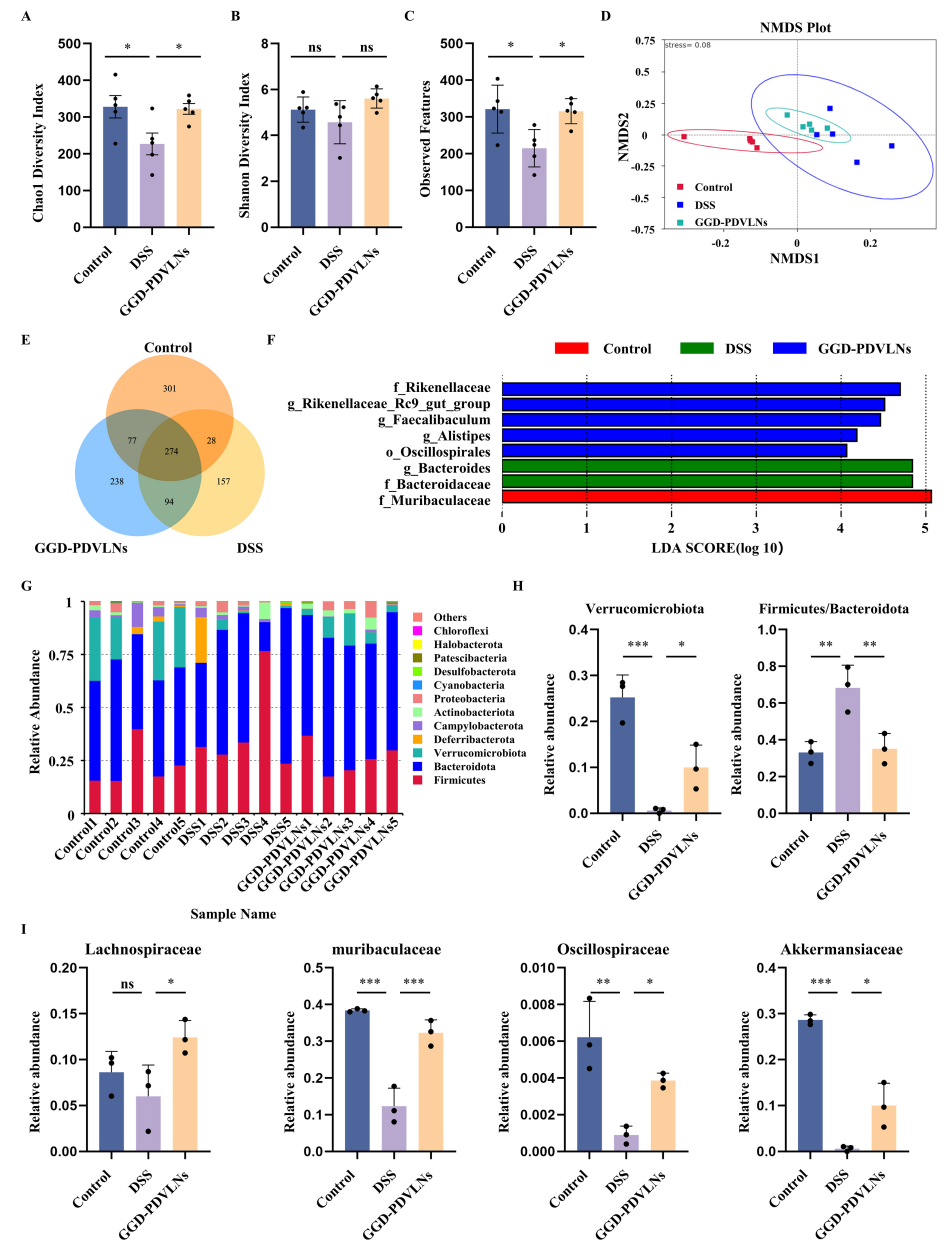
GGD-PDVLNs modulate gut microbiota diversity and increase the abundance of probiotics. (A) Estimation of microbial community observed ASVs richness; (B and C) Analysis of α-diversity (Chao1 and Shannon index); (D) NMDS analysis demonstrating the β-diversity of the intestinal microbiota; (E) Venn diagram of common and unique mouse species per group; (F) LEfSe analysis identifies species that differ significantly between groups of mice; (G) Relative abundance of the intestinal microbiota at the phylum level was determined based on its proportion relative to the total species count. *n* = 5; (H and I) Relative abundance of representative beneficial flora at both the phylum level and family level, *n* = 3. Data are shown as means ± SEM. Bar graphs were analyzed using one-way ANOVA. ^*^P < 0.05; ^**^P < 0.01; ^***^P < 0.001. GGD-PDVLNs: GeGen decoction-derived vesicle-like nanoparticles; ASVs: amplicon sequence variants; NMDS: non-metric multidimensional scaling; LEfSe: linear discriminant analysis effect size; SEM: standard error of the mean; ANOVA: analysis of variance; ns: no significance.

Given the critical involvement of gut microbiota dysbiosis in disease pathogenesis, we analyzed whether GGD-PDVLNs could modulate the gut microbial composition and restore gut microbial homeostasis. As shown in Figure 6A and B, the Chao1 index in the DSS group was significantly decreased, indicating a reduction in bacterial community abundance. In contrast, treatment with GGD-PDVLNs significantly increased the Chao1 index. The Shannon index showed the same trend as the Chao1 index, indicating that the GGD-PDVLNs restored the richness and diversity of the flora. A dramatic decrease in observed amplicon sequence variants (ASVs; DSS: 214 *vs*. control: 321) was reversed by GGD intervention [Figure 6C]. β-diversity assessment using Non-metric Multidimensional Scaling (NMDS) revealed distinct clustering patterns among experimental groups [Figure 6D]. The DSS group exhibited significant microbiota divergence from the controls, indicating that DSS induced dysbiosis. Treatment with GGD-PDVLNs effectively shifted microbial profiles toward the control cluster, indicating a partial restoration of commensal microbiota composition. The Venn diagram in Figure 6E shows that 274 common ASVs are shared among the three groups, indicating the presence of a core microbial community. The numbers of ASVs unique to the control group, DSS group, and GGD-PDVLNs were 301, 238, and 157, respectively, suggesting that the DSS treatment reduced species richness in mice, and that administration of GGD-PDVLNs reversed this effect. Linear Discriminant Analysis Effect Size (LEfSe) analysis in Figure 6F revealed significant enrichment of beneficial bacterial taxa - such as *Muribaculaceae*, *Rikenellaceae*, *Oscillospiraceae* - in the control group compared to the GGD-PDVLNs group. In contrast, *Bacteroidaceae* were significantly enriched in the DSS group.

To further investigate the effects of GGD-PDVLNs on the intestinal flora of mice, changes in the flora composition were analyzed at different taxonomic levels. The intestinal flora of mice in each group at the phylum level was mainly composed of Firmicutes and Bacteroidetes, and it has been reported that the ratio of Firmicutes to Bacteroidetes increased during UC^[41]^. This is in agreement with our findings that the ratio of Firmicutes to Bacteroidetes in the DSS group was significantly increased, and that treatment with GGD-PDVLNs alleviated this trend [Figure 6G and H]. Verrucomicrobiota is a common group of bacteria found in the intestinal tracts of animals and is a critical SCFA-producing phylum that was depleted in DSS mice. GGD-PDVLNs intervention reversed this decline [Figure 6G and H]. At the family level, the levels of beneficial bacteria including *Lachnospiraceae*, *Muribaculaceae*, *Oscillospiraceae* and *Akkermansiaceae* were decreased in the DSS group, which was reversed by the GGD-PDVLNs [Figure 6I]. At the genus level, the DSS group showed marked depletion of several beneficial genera, including *Akkermansia*, *Bifidobacterium*, *Dubosiella*, *Rikenellaceae_RC9_gut_group*, *Lachnospiraceae_NK4A136_group*, and *Parasutterella*. Notably, treatment with GGD-PDVLNs effectively restored the relative abundance of these probiotics [Supplementary Figure 4]. To elucidate the mechanism by which GGD-PDVLNs alleviate colitis through direct modulation of the gut microbiota, this study further investigated the interaction between GGD-PDVLNs and intestinal bacteria using an *in vitro* co-culture assay. The results demonstrated that fluorescently labeled GGD-PDVLNs could be internalized by the gut microbiota. Confocal microscopy images revealed that the fluorescent signals of the nanoparticles were localized within bacterial cells [Supplementary Figure 5]. This finding provides direct evidence that the gut microbiota can actively uptake GGD-PDVLNs, thereby influencing microbial composition. In conclusion, our results suggest that treatment with GGD-PDVLNs increases the levels of probiotic and SCFA-producing flora, bringing the flora composition of colitis mice closer to that of healthy mice.

### GGD-PDVLNs ameliorate colitis in mice mainly via gut microbiome regulation

**Figure 7:**
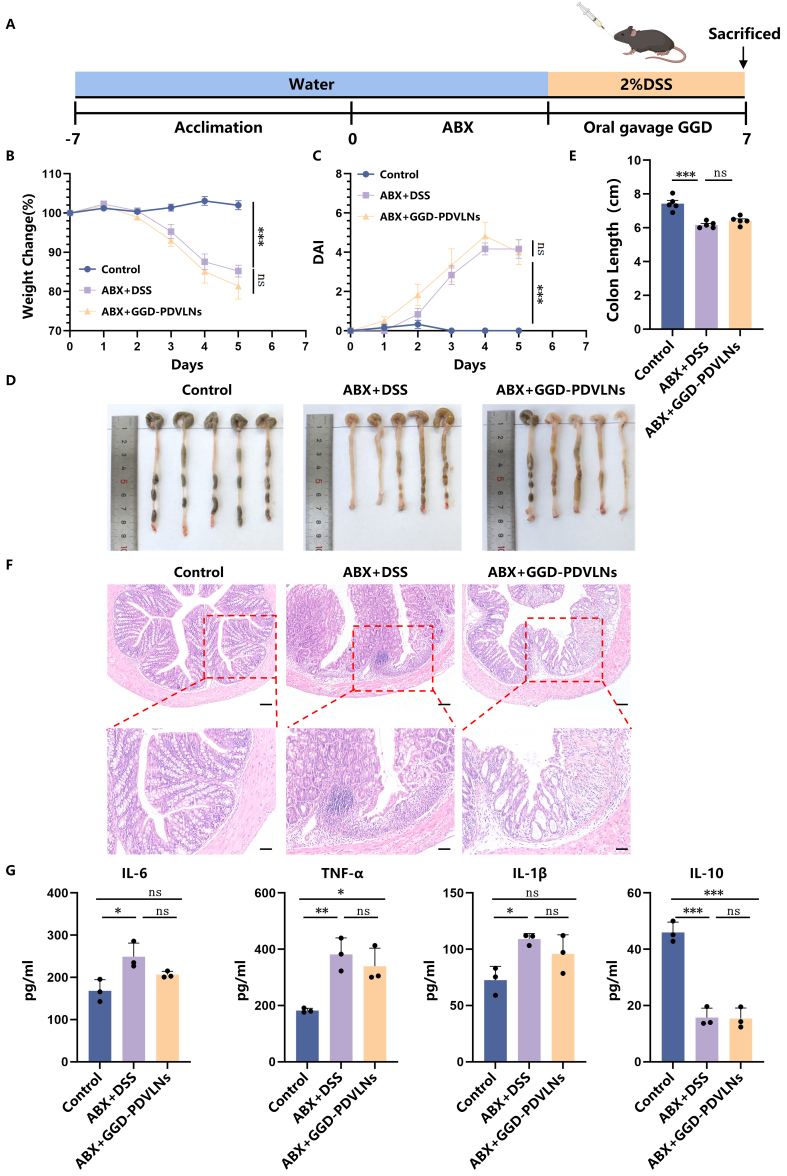
GGD-PDVLNs ameliorate colitis in mice mainly through gut microbiome regulation. (A) Establishment of a pseudo-germ-free model and administration of GGD-PDVLNs, Created in BioRender. Yijuan, H. (2026) https://BioRender.com/isfi061; (B) Change in body weight as a percentage of the weight on day zero; (C) DAI score; (D and E) Representative images of colons and average colon length from different groups, *n* = 5; (F) H&E-stained colon sections (100 and 50 μm, *n* = 3); (G) ELISA testing the expression profiles of IL-6, IL-1β, TNF-α, and IL-10 in colon tissue, *n* = 3. Data are shown as means ± SEM. Bar graphs were analyzed using one-way ANOVA, while line graphs were analyzed using two-way ANOVA. ^*^P < 0.05; ^**^P < 0.01; ^***^P < 0.001. GGD-PDVLNs: GeGen decoction-derived vesicle-like nanoparticles; DAI: disease activity index; H&E: hematoxylin and eosin; ELISA: enzyme-linked immunosorbent assay; IL: interleukin; TNF-α: tumor necrosis factor alpha; SEM: standard error of the mean; ANOVA: analysis of variance; ns: no significance.

To determine whether the therapeutic effects of GGD-PDVLNs are dependent on the gut microbiota, we established a model of antibiotic-mediated depletion of gut microbiota [Figure 7A]. The results demonstrated that GGD-PDVLNs lost their therapeutic efficacy following the elimination of gut microbiota, as evidenced by failure to alleviate body weight loss [Figure 7B], a persistent increase in DAI scores [Figure 7C], and no significant improvement in colon shortening (6.42 ± 0.22 cm) compared to the DSS group (6.17 ± 0.17 cm) [Figure 7D and E]. H&E staining further revealed that GGD-PDVLNs did not effectively ameliorate DSS-induced colon damage, as both the DSS group and the GGD-PDVLNs-treated mice exhibited marked inflammatory infiltration and crypt destruction in the colonic mucosa [Figure 7F]. This result suggests that GGD-PDVLNs exert their therapeutic effects mainly through the intestinal microbiota. Subsequent analysis of colonic inflammatory cytokine levels revealed that microbial ablation completely abolished the immunomodulatory effects of GGD-PDVLNs. As shown in Figure 7G and Supplementary Figure 6, under germ-free conditions, GGD-PDVLNs failed to suppress the expression of pro-inflammatory cytokines (IL-6, TNF-α, and IL-1β) at both the protein and mRNA levels. They failed to restore the production of the anti-inflammatory cytokine IL-10. These findings strongly indicate that the anti-inflammatory effects of GGD-PDVLNs are mediated through gut microbiota.

### Biosafety of orally administered GGD-PDVLNs

The biosafety profile of GGD-PDVLNs was rigorously evaluated through comprehensive *in vitro* and *in vivo* assessments. Hemolysis assays demonstrated excellent blood compatibility, with a hemolysis rate (HR) of 2.34% even at high concentrations (1.6 mg/mL; Supplementary Figure 7A and B). Major organs (heart, liver, spleen, lungs, and kidneys) of mice were collected for H&E staining. After oral administration of GGD-PDVLNs, there was no significant damage to the organs of mice [Supplementary Figure 7C]. Finally, the effects of GGD-PDVLNs on macrophage Raw 264.7 and Caco-2 cells were observed. The cell viability data showed no significant cytotoxicity of GGD-PDVLNs on these two cell types at a concentration of 100 μg/mL [Supplementary Figure 7D and E], demonstrating that GGD-PDVLNs have good biosafety.

## DISCUSSION

UC has emerged as a global health challenge, characterized by persistent damage to the colonic mucosal epithelium, which often leads to refractory disease courses and frequent relapse^[12,42]^. The current therapeutic paradigm primarily focuses on immunomodulation through regulation of cytokine balance. Yet, this approach fails to adequately address two fundamental pathological features: intestinal barrier dysfunction and microbial dysbiosis^[43]^. These limitations in both therapeutic efficacy and safety profiles underscore the urgent need for novel treatment strategies that target the gut ecosystem holistically. In this study, we demonstrated that GGD and its derived PDVLNs have significant therapeutic potential for UC by modulating the composition of the intestinal microbiota and host-microbe interactions. Furthermore, PDVLNs are the main active components through which GGD exerts anti-colitis effects. Comparative intervention studies revealed that PDVLNs-free GGD retained partial therapeutic activity, suggesting that GGDs containing other active substances, such as puerarin, also exerted some of their anti-colonic effects. In addition, Lu *et al*. had previously found that PDVLNs extracted from the fresh leaves and stems of GeGen had a mitigating effect on colitis^[44]^, suggesting that probably all PDVLNs from the whole plant source of GeGen have a beneficial effect on colitis.

Recent advances in plant-derived nanovesicle research have revealed that various plant species contain PDVLNs enriched with bioactive components, including proteins, lipids, and small RNAs, demonstrating remarkable therapeutic potential across multiple disease models^[9]^. Conventionally, PDVLNs have been isolated from fresh plants using homogenization and juice extraction methods. Alternatively, dried herbs have also served as viable sources, in which plant material is ground and incubated with PBS prior to differential centrifugation isolation. Notably, while traditional herbal tonics are usually prepared by boiling dried herbs in water^[45]^. Our results revealed that a large number of PDVLNs, rich in active substances such as proteins and lipids, and containing trace amounts of puerarin, remained stably present even in GGD after boiling decoctions. Previously, Yang *et al*. similarly detected heat-stable PDVLNs in herbal decoctions. Further compositional analysis indicated that PDVLNs were rich in sRNAs in the decoctions and showed potent anti-fibrotic and anti-inflammatory effects^[46]^. This suggests that herbal decoctions may be a potential source for obtaining and isolating PDVLNs, offering a new perspective for exploring active ingredients in Chinese medicine.

Previous studies by Zhan *et al*. and Zhang *et al*. successfully isolated and characterized PDVLNs from fresh GeGen, revealing well-defined nanoparticles with a size distribution ranging from 119 to 209.7 nm and characteristic vesicular morphology, as confirmed by TEM^[47,48]^. Our results were consistent with theirs, indicating that the GGD-PDVLNs are also well preserved in the dried herbs. In the treatment of UC, oral administration offers significant advantages over intravenous and intraperitoneal routes. It is not only more convenient but also associated with improved safety, greater patient compliance, and a higher likelihood of reaching the target site to exert therapeutic effects. Our study demonstrates that GGD-PDVLNs exhibit remarkable GI stability, making them suitable for oral administration in the treatment of UC. This characteristic aligns with other PDVLNs derived from turmeric^[25]^, tea^[21]^ and ginger^[28]^, all of which show similar resistance to digestive degradation. The exceptional stability of these nanoparticles can be attributed to their unique lipid-rich membrane composition, which serves as a protective barrier against GI enzymes. The membrane of PDVLNs consists of lipids, which play an important role in maintaining their stability. Our lipidomics analysis revealed a distinctive lipid signature in GGD-PDVLNs, prominently characterized by TG, DG, Cer, PE, and PC. This composition, notably enriched in neutral lipids and sphingolipids, differs markedly from the polar lipid-dominated profiles [e.g., Phosphatidic Acid (PA), Phosphatidylinositol(PI), PE, PC, Digalactosyldiacylglycerol (DGDG)] typically reported in fresh plant materials or chloroplast-derived vesicles^[9,10]^. While similar shifts toward neutral and sphingolipids have been observed in PDVLNs from other herbal decoctions^[49]^, emerging evidence suggests that this lipid profile may be intrinsically associated with the plant species itself. Notably, lipidomic studies of PDVLNs isolated from fresh, unboiled Pueraria lobata have shown comparable enrichment in TG, DG, Cer, and related sphingolipids^[50]^, indicating that this lipid composition is more likely a fundamental biochemical trait of this medicinal plant. The traditional decoction process may help selectively extract or stabilize this native lipid repertoire, thereby potentially altering the relative abundance of specific lipid species.

Proteomic analysis reveals that PDVLNs contain a diverse array of proteins that likely contribute to their biological functions. Our investigation of GGD-PDVLNs, together with previous findings by Yang *et al*.^[51]^, demonstrates that these nanoparticles are predominantly enriched in cytoplasmic proteins, with a relatively lower abundance of membrane-associated proteins. Notably, the preservation of protein composition does not necessarily indicate functional dependence. Indeed, the extraction of GGD-PDVLNs via high-temperature decoction suggests that their biological activity is not protein-dependent.

Plant extracts and phytochemicals have been the primary focus in the treatment of human diseases, including UC, and existing studies have demonstrated that PDVLNs also contain such bioactive compounds. For example, turmeric-derived PDVLNs contain anti-inflammatory curcuminoids^[25]^, ginger-derived PDVLNs contain 6-gingerol and 6-shogaol^[28]^, and tea-derived PDVLNs are enriched in polyphenols and flavonoids^[21]^. The content of these phytochemicals varied significantly across plant species, ranging from 3 to 150 μg/mg of PDVLNs. In contrast to the findings of Zhang *et al*.^[48]^, our study demonstrated a low geraniol content of geraniols of 35.4 μg ± 1.2 μg/mg in GGD-PDVLNs. Notably, while previous studies have reported effective doses of puerarin for the treatment of UC ranging from 50 to 200 mg/kg^[13,15]^, our subsequent experiments employed a substantially lower GGD-PDVLN dosage of 15 mg/kg - corresponding to only 0.531 mg/kg of puerarin, which is significantly below the dose typically required for therapeutic efficacy of puerarin alone. This suggests that the therapeutic efficacy of GGD-PDVLNs may primarily derive from other active components such as proteins, lipids or sRNAs, or that the nanoparticle formulation enhances the bioavailability and delivery efficiency of puerarin, thereby enabling comparable therapeutic effects at dramatically reduced doses.

Furthermore, our study revealed that GGD-PDVLNs exerted significant modulatory effects on the gut microbiota in a microbiota-dependent manner. The therapeutic efficacy was completely abolished in pseudo germ-free mice, demonstrating an essential role of gut microbes in mediating the beneficial effects of GGD-PDVLNs. The entire intestinal flora system is considered a “microbial organ” that contributes to various physiological processes, including the metabolism of fermented indigestible dietary fiber, anaerobic metabolism of peptides and proteins, and immune system regulation^[52]^, all of which play an important role in the development of UC. In patients with UC, this is often accompanied by decreased microbial diversity and stability, and by an expansion of Proteobacteria, including *Enterobacteriaceae* and certain *Bacteroidetes*^[53]^. The host and gut flora interact to produce a wide range of metabolites, including SCFAs and bile acids, which help modulate intestinal inflammation^[54]^. Therefore, restoring gut homeostasis in UC is also one of the key pathways to treating UC.

Our findings demonstrate that GGD-PDVLNs restore gut microecological homeostasis in colitis mice by selectively enriching key SCFA-producing probiotics. These include *Lachnospiraceae, Muribaculaceae, Oscillospiraceae, Akkermansiaceae,* and genera such as *Bifidobacterium* and *Rikenellaceae_RC9_gut_group* - all known for reinforcing intestinal barrier integrity, producing anti-inflammatory metabolites, and maintaining immune balance^[55-58]^. By reversing DSS-induced dysbiosis, GGD-PDVLNs disrupt the pro-inflammatory cascade and promote mucosal healing.

GGD-PDVLNs are enriched with bioactive components, including lipids and proteins. They may act as specific signaling molecules or metabolic substrates that are selectively utilized by beneficial bacteria, thereby exerting a prebiotic-like effect that promotes the proliferation of probiotics. As demonstrated in our study, GGD-PDVLNs significantly reduced the secretion of pro-inflammatory cytokines and enhanced the expression of tight junction proteins, thereby repairing the compromised intestinal barrier. This improvement is critical, as an anti-inflammatory and structurally intact intestinal environment effectively suppresses the overgrowth of opportunistic pathogens and creates a stable ecological niche for the colonization and expansion of beneficial bacteria, thereby indirectly promoting a beneficial shift in the microbial community^[19,20]^. Following GGD-PDVLNs intervention, the abundance of SCFA-producing bacteria, such as *Lachnospiraceae* and *Akkermansiaceae*, was markedly restored. The SCFAs produced by these bacteria serve as a primary energy source for colonic epithelial cells, further strengthening the barrier function and directly modulating immune cell activity^[59,60]^. In summary, GGD-PDVLNs likely exert their therapeutic effects via a synergistic “microbiota-gut-immune” axis. The nanoscale carriers facilitate the targeted delivery of active components to the gut, directly and indirectly remodeling the dysregulated microbiota. The enriched probiotics and their metabolites subsequently improve barrier integrity and immune homeostasis, ultimately alleviating colitis. This mechanistic framework highlights the significant potential of plant-derived nanoparticles as ecological modulators for the treatment of IBD.

While this study demonstrates that GGD-PDVLNs alleviate colitis through microbiota-dependent mechanisms, several limitations should be acknowledged. Although our depletion experiments strongly indicate that GGD-PDVLNs are the primary active components, the ultracentrifugation method used to prepare PDVLN-free GGD may have co-removed other macromolecular complexes, potentially contributing to its attenuated efficacy. While our sequential centrifugation protocol (1,000-10,000 × *g*) effectively eliminates dead cells and large debris, ultracentrifugation alone may not yield highly pure GGD-PDVLNs. The boiling step during decoction preparation denatures and inactivates most soluble proteins, thereby reducing the risk of functional protein contamination. Future studies could employ more specific isolation techniques, such as immunoaffinity capture, size‑exclusion chromatography, or sucrose density gradient centrifugation, to obtain higher‑purity GGD-PDVLNs and more precisely delineate the contribution of PDVLNs per se. Secondly, although our depletion and isolation experiments demonstrated the necessity and sufficiency of GGD-PDVLNs for the therapeutic efficacy of the GGD, a direct comparative assessment of the therapeutic potency between the full decoction and the isolated nanoparticles was not conducted. Such a comparison is essential to quantify any potential advantages conferred by the extraction and purification process, which represents a critical consideration for future translational development. While we identified specific probiotic taxa that increased following GGD-PDVLNs treatment, the precise molecular mechanisms by which GGD-PDVLNs modulate these bacterial populations remain unclear. Whether specific lipids, proteins, or RNAs carried by GGD-PDVLNs are responsible for shaping the microbial community warrants further investigation using targeted compositional analysis and microbial functional assays. Lastly, the current findings are based on a murine DSS-induced colitis model. The translational relevance of these results to human UC requires validation in future clinical studies.

In summary, this study investigated the therapeutic potential of PDVLNs derived from traditional Chinese herbal decoctions, using GGD as a representative example, and revealed their anti-inflammatory effects in intestinal disorders that extend beyond those attributable to known secondary metabolites. Compared to animal-derived extracellular vesicles, PDVLNs offer superior advantages, including broader availability, higher production yields, lower preparation costs, reduced immunogenicity, and exceptional GI stability that enables direct oral delivery to inflamed intestinal sites. These findings position herbal decoction-derived PDVLNs as promising functional components for intestinal health maintenance, serving as a novel bridge between phytotherapy and microbiome science. Importantly, they establish a groundbreaking therapeutic strategy for IBDs through ecological restoration of the gut microenvironment. This research provides both theoretical and practical foundations for innovative therapeutic strategies for UC rooted in the modernization of traditional medicine.

## DECLARATIONS

### Availability of data and materials

The raw data supporting the findings of this study, including microbiome sequencing data, are available within this Article and its Supplementary Materials. Further datasets generated and analyzed during the current study are available from the corresponding authors upon reasonable request.

### AI and AI-assisted tools statement

We employed AI-assisted tools to perform language editing and polishing on selected text passages, aiming to enhance clarity and conciseness while rigorously maintaining scientific accuracy. The use of these technologies promotes transparency and trust among authors, readers, reviewers, editors, and contributors, and facilitates compliance with the terms of use of the relevant tools or technologies.

### Copyright

© The Author(s) 2026.
