# Supplementary material for: Vesicle-like nanoparticles extracted from Pueraria lobata decoction alleviate colitis by modulating the intestinal microbiota
Source: Extracell Vesicles Circ Nucl Acids. 2026 Feb 12;7(1):234–58. doi: 10.20517/evcna.2025.134 (PMC13074279; doi:10.20517/evcna.2025.134)
Supplement: Supplementary file 1 [file evcna-7-1-234-SupplementaryMaterials.pdf]

Supplementary Materials

Vesicle-like nanoparticles extracted from *Pueraria lobata* decoction alleviate colitis by modulating the intestinal microbiota

Cai-Xiao Liu[#], Yi-Juan Han[#], Na Zhao[#], Qiao-Ning Wang, Run-Run Wan, Ting-Ting Cao, Xi He, Cheng-Hu Hu, Cheng-Biao Hu, Zhang Yuan

Xi'an Key Laboratory of Stem Cell and Regenerative Medicine, Institute of Medical Research, Northwestern Polytechnical University, Xi'an 710072, Shaanxi, China.
[#]These authors contributed equally to this work.

Correspondence to: Prof. Cheng-Hu Hu, Dr. Cheng-Biao Hu, Prof. Zhang Yuan, Xi'an Key Laboratory of Stem Cell and Regenerative Medicine, Institute of Medical Research, Northwestern Polytechnical University, Xi'an 710072, Shaanxi, China. E-mail: chenghu@nwpu.edu.cn; hcbhappy@nwpu.edu.cn; yuanzhang1993@nwpu.edu.cn

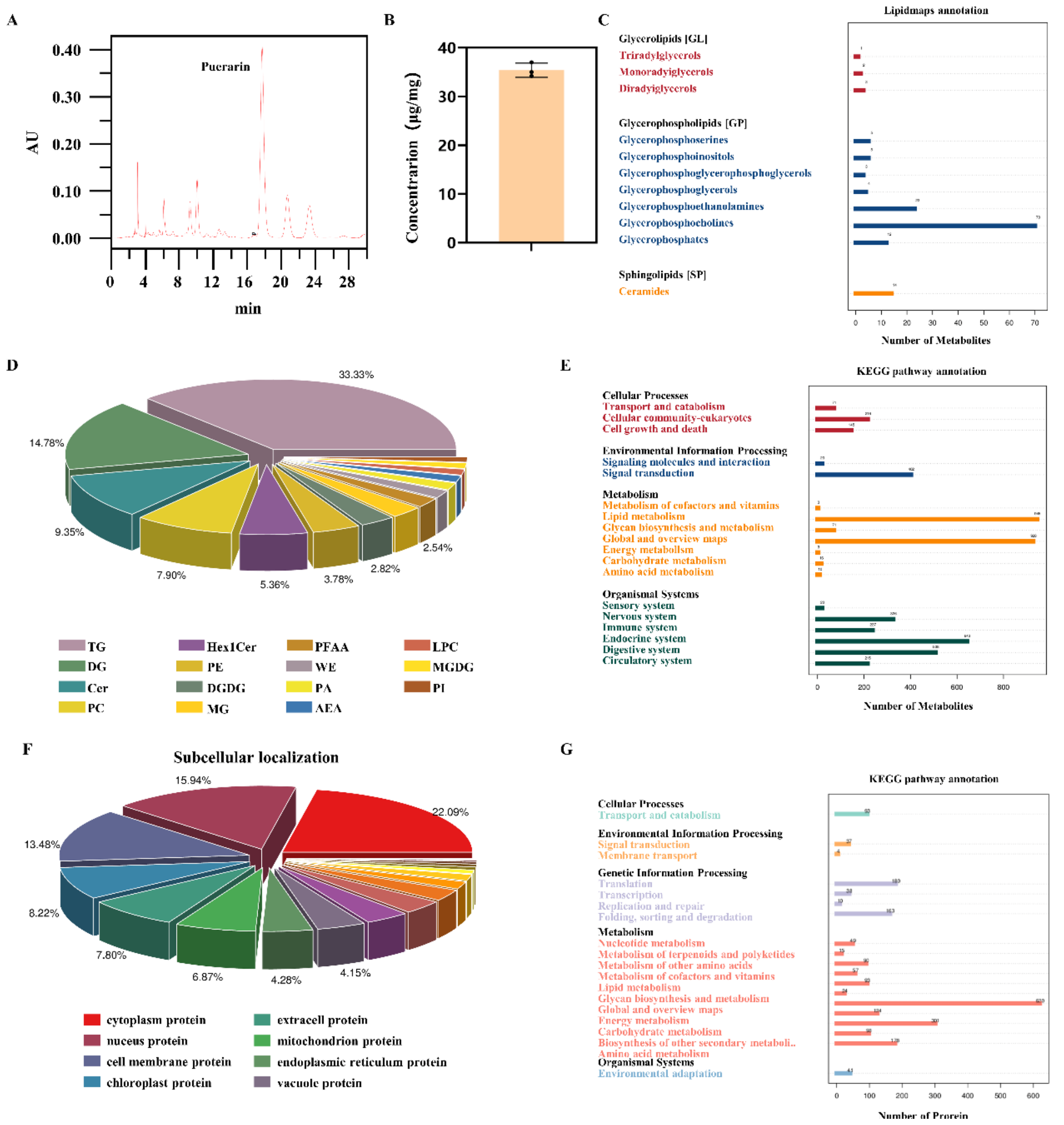

Supplementary Figure 1. Identification and analysis of active substances in GGD-PDVLNs. (A) The HPLC spectrum of puerarin in GGD-PDVLNs; (B) Quantitative analysis of puerarin in GGD-PDVLNs, $n = 3$; (C) LIPID MAPS classification notes of GGD-PDVLNs; (D) lipid profiles of GGD-PDVLNs; (E) KEGG annotation of lipids in GGD-PDVLNs; (F) Protein subcellular localization analysis; (G) KEGG annotated of proteins in GGD-PDVLNs. Data shown as means ± SEM.

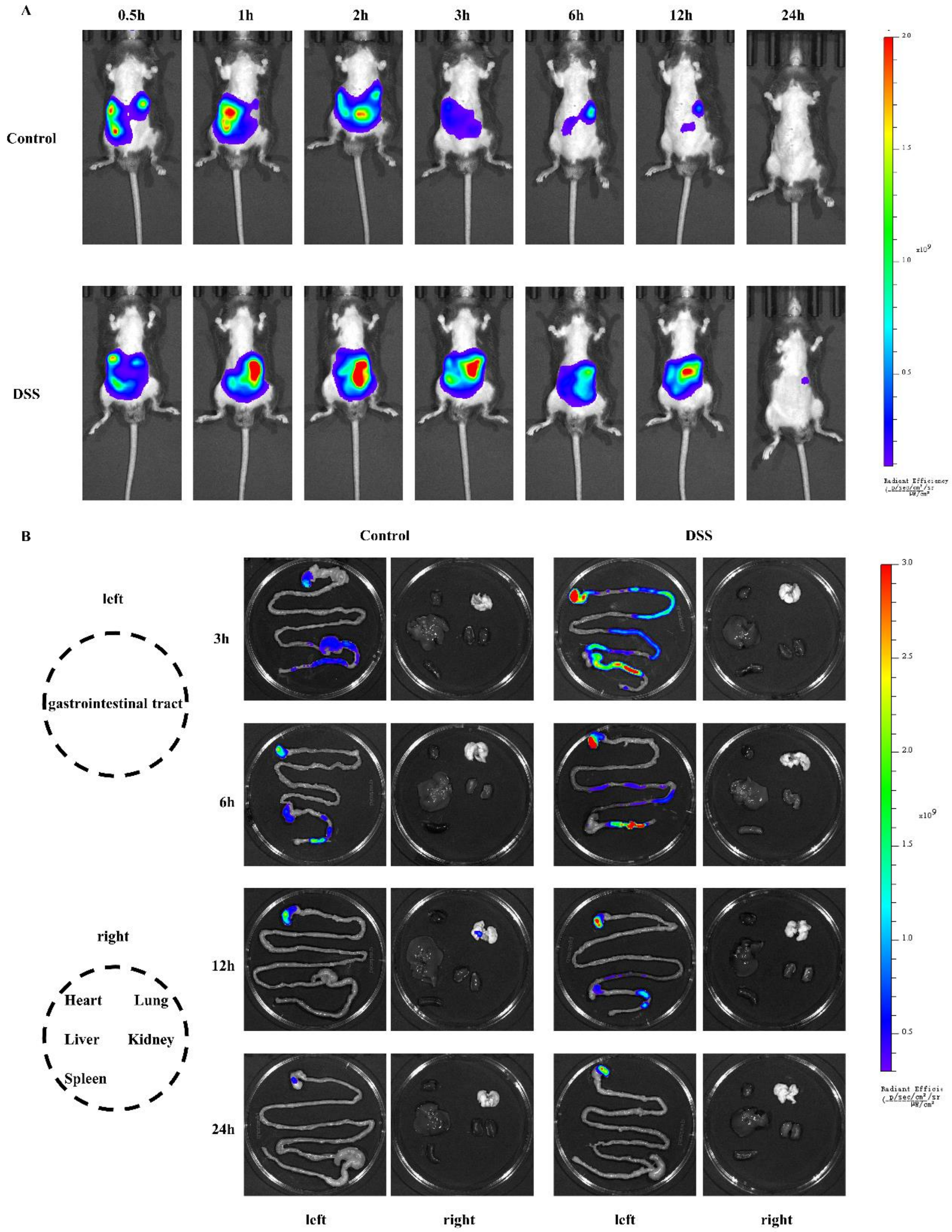

Supplementary Figure 2. GGD-PDVLNs can remain in the inflammatory colon. (A) Whole body imaging of healthy and colitis mice after oral administration of GGD-PDVLNs; (B) The distribution of DiR-labeled GGD-PDVLNs in the gastrointestinal tract, heart, liver, spleen, lung, and kidney at different time points, $n = 3$.

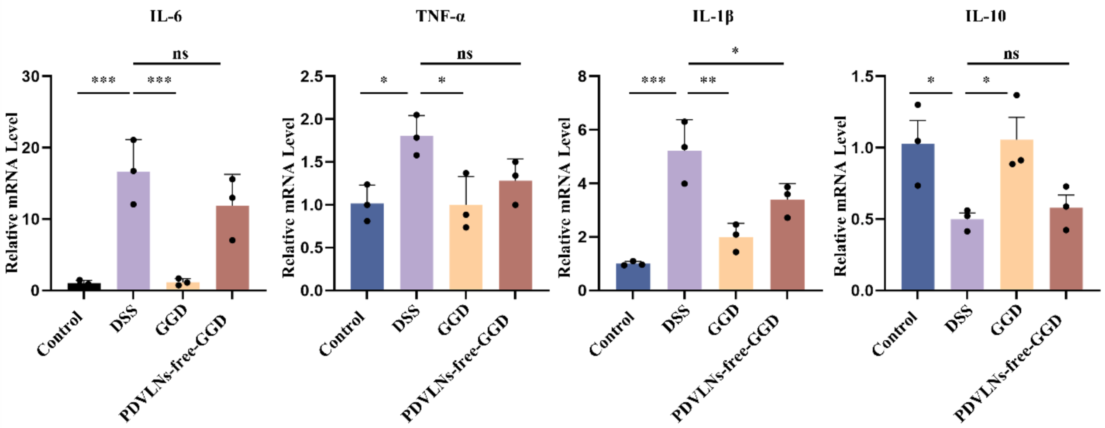

Supplementary Figure 3. RT-qPCR detecting the levels of IL-6, IL-1β, TNF-α, and IL-10 in colon tissue, n=3. Data shown as means ± SEM. Bar graphs were analyzed using one-way ANOVA, ns = no significance, *P < 0.05, **P < 0.01, ***P < 0.001.

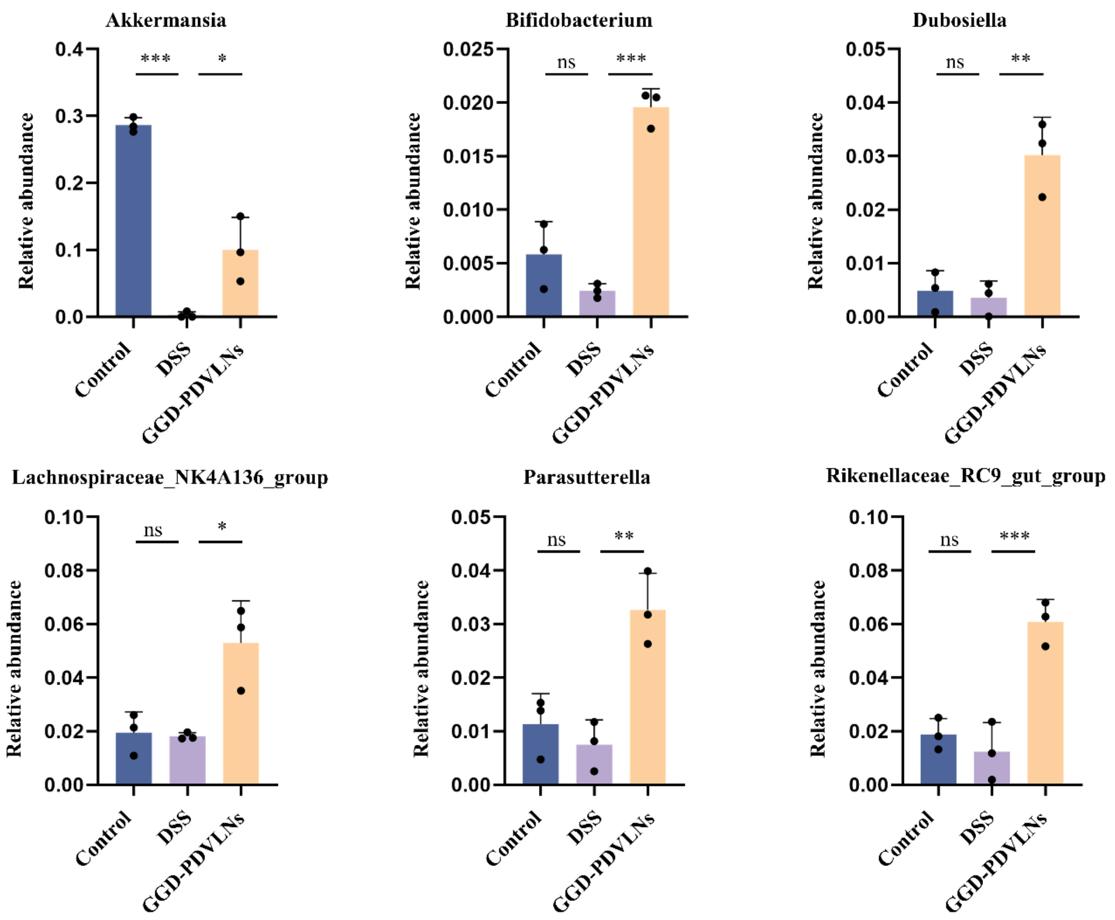

Supplementary Figure 4. Relative abundance of representative beneficial flora at the genus and family levels, $n = 3$. Data shown as means ± SEM, ns = no significance. Bar graphs were analyzed using one-way ANOVA, $*P < 0.05$, $**P < 0.01$, $***P < 0.001$.

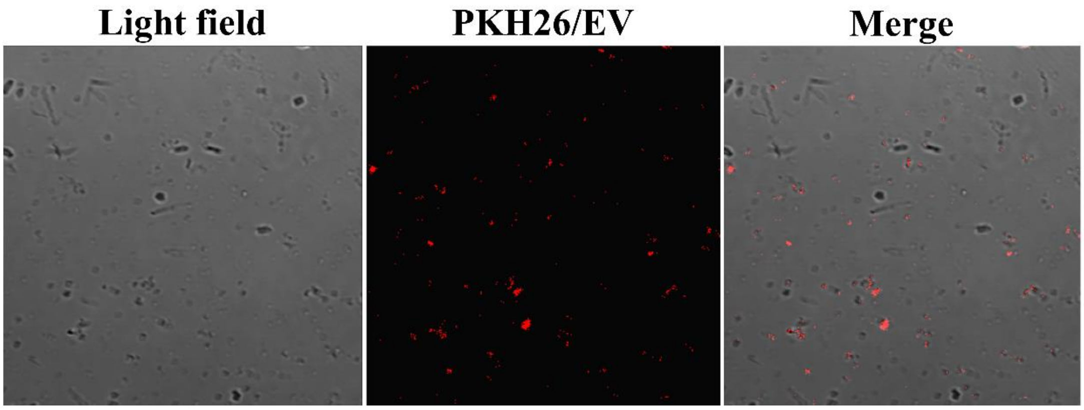

Supplementary Figure 5. Fluorescence microscopy images showing in vitro uptake of PKH26-labeled GGD-PDVLNs by gut bacteria (scale bar: 5 μm).

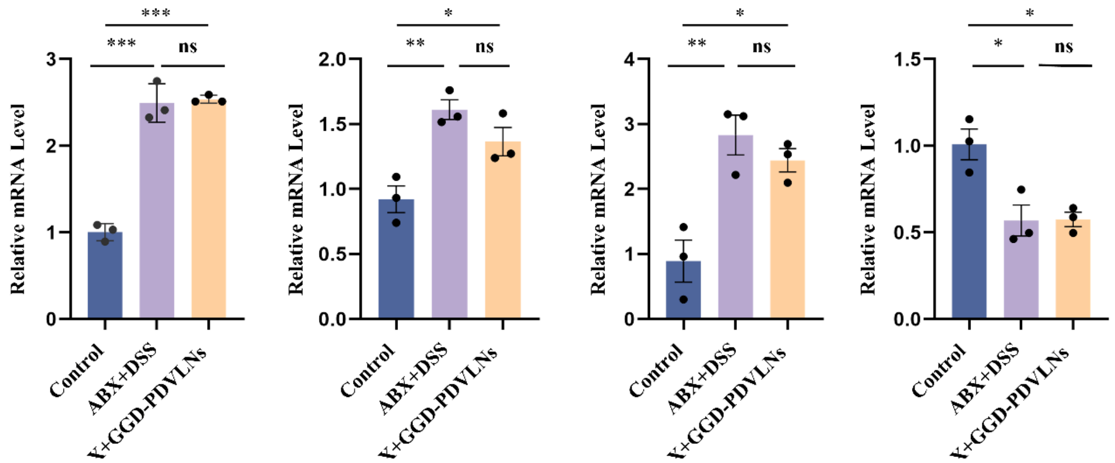

Supplementary Figure 6. RT-qPCR detecting the levels of IL-6, IL-1β, TNF-α, and IL-10 in colon tissue, $n = 3$. Data shown as means ± SEM. Bar graphs were analyzed using one-way ANOVA, ns = no significance, $^{*}P < 0.05$, $^{**}P < 0.01$, $^{***}P < 0.001$.

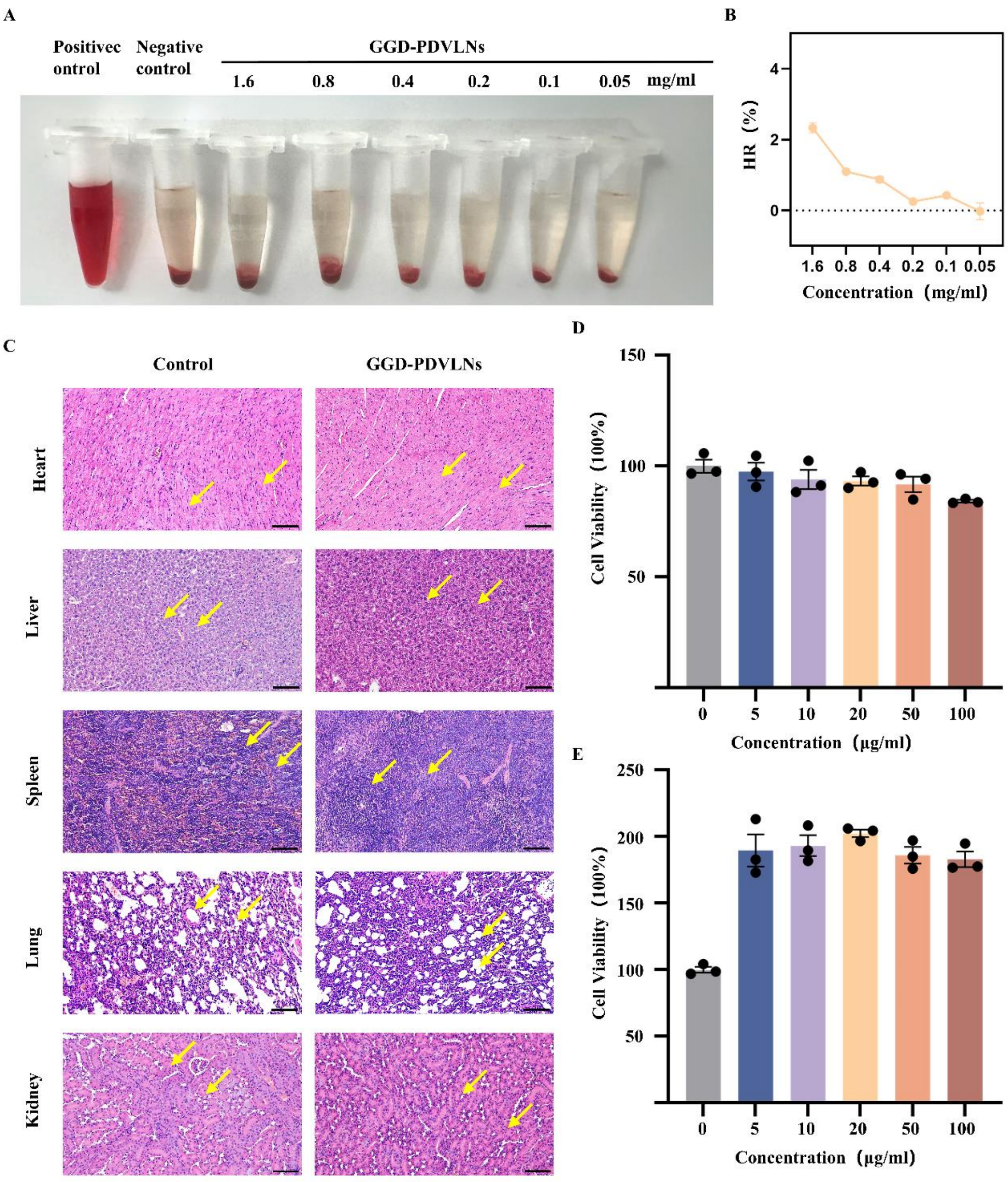

Supplementary Figure 7. Biosafety of orally administered GGD-PDVLNs. (A and B) Detection of haemolysis rate after 1h incubation of different concentrations of GGD-PDVLNs with fresh blood from mice; (C) H&E staining of major organs (heart, liver, spleen, lung, kidney) in mice. The arrows in the image point to the characteristic structures of the respective tissues and organs, including myocardial fibers, hepatic lobules, red pulp/white pulp, alveoli, and renal glomeruli; (D and E) Cell activity of RAW264.7 and Caco-2 cells after 24 h incubation with different concentrations of GGD-PDVLNs. *n* = 3. Data shown as means ± SEM.

Supplementary Table 1. Primer sequences applied in the paper

Gene	Forward	Reverse
GAPDH (mouse)	5′-GGTTGTCTCCTGCGACTTCA-3′	5′-TGGTCCAGGGTTTCTTACTCC-3′
IL-6 (mouse)	5′-AGTTGCCTTCTTGGGACTGA-3′	5′-CAGAATTGCCATTGCACAAC-3′
IL-1β (mouse)	5′-TTCAAGGGGACATTAGGCAG-3′	5′-TGTGCTGGTGCTTCATTCAT-3′
TNF-α (mouse)	5′-CTCAGCGAGGACAGCAAGG-3′	5′-AGGGACAGAACCTGCCTGG-3′
IL-10 (mouse)	5′-AGCCTTATCGGAAATGATCCAG-3′	5′-GGCCTTGTAGACACCTTGGT-3′
